# Efficacy of a Text Message-Based Smoking Cessation Intervention for Young People: A Cluster Randomized Controlled Trial

**DOI:** 10.2196/jmir.2636

**Published:** 2013-08-16

**Authors:** Severin Haug, Michael P Schaub, Vigeli Venzin, Christian Meyer, Ulrich John

**Affiliations:** ^1^Swiss Research Institute for Public Health and Addictionan associated Institute of the University of ZurichZurichSwitzerland; ^2^Cantonal Office for Secondary Education ZurichZurichSwitzerland; ^3^Institute of Social Medicine and PreventionUniversity Medicine GreifswaldGreifswaldGermany

**Keywords:** smoking cessation, text messaging (SMS), young people, school, students

## Abstract

**Background:**

Smoking prevalence remains high, particularly among adolescents and young adults with lower educational levels, posing a serious public health problem. There is limited evidence of effective smoking cessation interventions in this population.

**Objective:**

To test the efficacy of an individually tailored, fully automated text messaging (short message service, SMS)–based intervention for smoking cessation in young people.

**Methods:**

A 2-arm cluster randomized controlled trial, using school class as the randomization unit, was conducted to test the efficacy of the SMS text messaging intervention compared to an assessment-only control group. Students who smoked were proactively recruited via online screening in vocational school classes. Text messages, tailored to demographic and smoking-related variables, were sent to the participants of the intervention group at least 3 times per week over a period of 3 months. A follow-up assessment was performed 6 months after study inclusion. The primary outcome measure was 7-day smoking abstinence. Secondary outcomes were 4-week smoking abstinence, cigarette consumption, stage of change, and attempts to quit smoking. We used regression models controlling for baseline differences between the study groups to test the efficacy of the intervention. Both complete-case analyses (CCA) and intention-to-treat analyses (ITT) were performed. Subgroup analyses were conducted for occasional and daily smokers.

**Results:**

A total of 2638 students in 178 vocational school classes in Switzerland participated in the online screening. Overall, 1012 persons met the inclusion criteria for study participation, and 755 persons (74.6%) participated in the study (intervention: n=372; control: n=383). Of the 372 program participants, 9 (2.4%) unsubscribed from the program during the intervention period. Six-month follow-up data were obtained for 559 study participants (74.0%). The 7-day smoking abstinence rate at follow-up was 12.5% in the intervention group and 9.6% in the control group (ITT: *P*=.92). No differences between the study groups were observed in 4-week point prevalence abstinence rates. The decrease in the mean number of cigarettes smoked per day from baseline to follow-up was higher in the intervention group than in the control group (ITT: *P*=.002). No differences between the groups were observed in stage of change (ITT: *P*=.82) and quit attempts (ITT: *P*=.38). The subgroup analyses revealed lower cigarette consumption in both occasional and daily smokers in the intervention group compared to the control group. Occasional smokers in the intervention group made more attempts to quit smoking than occasional smokers in the control group.

**Conclusions:**

This study demonstrated the potential of an SMS text message–based intervention to reach a high proportion of young smokers with low education levels. The intervention did not have statistically significant short-term effects on smoking cessation; however, it resulted in statistically significant lower cigarette consumption. Additionally, it resulted in statistically significant more attempts to quit smoking in occasional smokers.

**Trial Registration:**

International Standard Randomized Controlled Trial Number (ISRCTN): 19739792; http://www.controlled-trials.com/ISRCTN19739792 (Archived by WebCite at http://webcitation.org/6IGETTHmr).

## Introduction

Tobacco use is a major cause of the global disease burden and is the single most preventable cause of death in the world [1]. A survey of 15- and 16-year-old adolescents covering 36 European countries revealed that the smoking prevalence rate of 28% having used cigarettes during the past 30 days has remained stable over the past 4 years [2]. Smoking continues to be a serious problem, particularly in adolescents and young adults with lower education levels [3].

There is limited evidence of smoking cessation interventions demonstrating efficacy in young people [4,5]. The 2006 Cochrane Review for smoking cessation interventions for those younger than 20 years identified only 15 trials of sufficient quality, of which only 1 [6] found statistically significant evidence of an intervention effect [4]. The authors acknowledged that there is a need for well-designed, adequately powered trials of cessation interventions. The authors concluded that complex approaches, including elements sensitive to stage of change, achieved moderate long-term success, whereas the efficacy of psychosocial and pharmacological interventions could not be demonstrated. A more recent but less systematic review from 2008 [5] suggested that delivering smoking cessation programs for youth in contexts that are geared to youth, interventions addressing cognitive behavioral, motivational and social influence contents, and programs with at least 5 sessions were most effective. Since the publication of these reviews, additional randomized controlled trials of adolescent smoking interventions have been reported, from which 2 found a treatment effect at 6-month follow-up: Pbert et al [7] provided brief counseling by the pediatric provider followed by 1 visit and 4 telephone calls by older peer counselors; Peterson et al [8] provided proactive telephone counseling of high school juniors.

Beyond intervention effectiveness, intervention reach and retention are major challenges of smoking cessation interventions in young people [9,10]. Reaching a large proportion of adolescent smokers has been difficult. Less than 50% of smokers are typically recruited in school-based smoking cessation programs [4,11]. However, a large reach is essential for the efficacy of an intervention at the population level. For a large reach, proactive recruitment strategies are needed that address all persons among a given target population. All smokers should receive the invitation to take part in smoking cessation. Such recruitment, in combination with low-threshold interventions, seems promising [7,8].

Mobile phone text messaging (short message service, SMS) is very popular among adolescents and young adults and has the potential to deliver smoking cessation support to large proportions of the population. Among 12- to-19-year-old adolescents from Switzerland, 98% owned a mobile phone in 2010; use of the mobile phone was the most frequent leisure time activity in this population group [12]. Reading and sending SMS text messages were the most frequent activities when using a mobile phone [12].

By using expert system technology that provides information based on individual demographic- or smoking-related characteristics, electronic communication technology can be a viable time- and cost-saving alternative to interpersonal counseling [13]. SMS text messaging provides an opportunity for individualized and interactive information delivery that may easily be accessed, independent of time and place. A recent Cochrane Review including 5 randomized or quasi-randomized studies revealed an overall long-term benefit of mobile phone interventions for smoking cessation in adults [14], although there was a high level of statistical heterogeneity in the pooled results. A large, methodologically sound trial was conducted in Great Britain to test the efficacy of SMS text message-based smoking cessation interventions in adults motivated to quit smoking [15]. Within this study, smokers who intended to quit within the subsequent month received motivational messages and behavioral-change support over a period of 26 weeks. The messages were matched to participants’ demographic and smoking-related characteristics gathered at baseline. Additionally, participants could request instant messages aimed at craving or lapse situations. The program significantly improved smoking cessation rates at 6 months compared to a control group that received text messages unrelated to quitting (9% vs 4%, respectively).

To date, neither randomized controlled trials testing the efficacy of smoking cessation interventions employing SMS text messaging in adolescents and young adults nor trials testing the efficacy of SMS text message interventions in proactively recruited smokers have been reported. In 3 pilot studies in which young adult smokers, irrespective of their motivation to quit, were proactively invited to an SMS text message-based smoking cessation intervention, high participation and retention proportions were achieved [16-18].

Within the present cluster randomized trial, we tested the efficacy of an SMS text message-based intervention for smoking cessation in a sample of proactively recruited students with varying motivation to quit. Vocational school students were chosen as the target population because smoking prevalence rates in this subgroup of adolescents and young adults with heterogeneous educational levels are high [3].

## Methods

### Study Design

A 2-arm cluster randomized controlled trial (ISRCTN: 19739792 assigned on May 20, 2011) was conducted to test the efficacy of the program SMS-COACH, an SMS text message-based intervention for smoking cessation in adolescents and young adults, compared to an assessment-only control group. The trial was undertaken in Switzerland, and participants were recruited between October 2011 and May 2012. The 6-month follow-ups were conducted between April and December 2012; the study protocol was published on January 19, 2012 [19]. Students in vocational schools were proactively invited to participate, irrespective of their intention to quit. The smoking cessation text messages were primarily based on the Health Action Process Approach (HAPA) [20] and included cognitive behavioral and motivational components according to this model. Text messages were sent to the participants over a period of 3 months and were tailored according to data gathered at baseline and a weekly SMS text message assessment. At the 6-month follow-up, we expected a higher 7-day point prevalence smoking abstinence rate in students in the intervention group compared to students in the assessment-only control group. Secondary outcome measures were 4-week point prevalence smoking abstinence, the number of cigarettes smoked per day, stage of change, and number of attempts to quit smoking. The study protocol was approved by the Local Ethics Committee of the Canton of Zurich, Switzerland (date of approval: March 15, 2011; No: KEK-StV-Nr. 05/11). The trial was executed in compliance with the Helsinki Declaration.

The study was implemented as described in the study protocol [19] with the following modifications: (1) because of smaller class sizes than expected and time restrictions, we could not reach the targeted sample size of 910 study participants, but enrolled 755 study participants; (2) self-efficacy for smoking cessation could not be assessed at follow-up and used as a secondary outcome measure because the rating scale to assess this variable [20] could not be applied in the telephone interviews conducted at follow-up; and (3) nicotine dependence could not be calculated for occasional smokers using the Heaviness of Smoking Index [21]. Therefore, we used number of cigarettes smoked per day as an indicator of nicotine dependence and as an outcome variable.

### Participant Recruitment and Baseline Assessment

Smoking students were recruited at vocational schools in Switzerland. Contact teachers for addiction prevention or headmasters of 57 vocational schools in German-speaking regions of Switzerland were invited to enroll some of their classes in a study testing the efficacy of an SMS text message–based smoking cessation program. Teachers from the 24 participating vocational schools scheduled 1 school hour per class for screening of eligibility criteria, study information, baseline assessments, and program registration. Study participants were recruited by study assistants (graduate students of psychology). The study assistants invited all students from a school class to participate in an online health survey during a regular school lesson reserved for health education. They informed the students that some people would be invited to participate in a study testing the efficacy of an SMS text message intervention for health promotion. To decrease reporting bias, the study assistants did not provide more information about the purpose of the study before the screening of eligibility criteria was completed.

Afterwards, the students were invited to complete an online screening. The screening included the assessment of demographic data, alcohol consumption, weekly physical activity, smoking status, and ownership of a mobile phone. Inclusion criteria for study participation were (1) daily or occasional cigarette smoking (at least 4 cigarettes in the preceding month and at least 1 cigarette during the preceding week), and (2) ownership of a mobile phone. Subsequently, eligible persons were informed by the online program about the aim of the study, the intervention arms, assessments, reimbursement, and data protection. Study information was provided online and in paper form by the study assistants. The equivalent of €8 was offered as reimbursement to all study participants for participation at the 6-month follow-up assessment. Additionally, the equivalent of €0.80 was offered as reimbursement to the participants of the intervention group for each SMS text message response to the weekly SMS text message assessments in the program. After receiving informed consent online, all study participants were invited to choose a username and to provide their mobile phone number. Subsequently, the following smoking-related variables were assessed: stage of change, number of cigarettes smoked per day, past quit attempts, and age of smoking onset. Afterwards, study participants of the intervention group received further information about the operation of the program. Control group participants were informed that they were assigned to the control group and could not participate in the SMS text message program.

### Randomization and Allocation Concealment

To avoid spillover effects within school classes, we used cluster randomization with school class as the randomization unit. Because of the heterogeneity of students in the different vocational schools (ie, gender or course of study), we used separate randomization lists for each vocational school (stratified randomization). Furthermore, to approximate equality of sample sizes in the study groups, we used block randomization with computer-generated, randomly permuted blocks of 4 cases [22].

The study assistants who conducted the baseline assessment in the vocational schools were blinded concerning group allocation for each of the school classes. Additionally, group allocation was not released to study participants until they provided informed consent, username, mobile phone number, and baseline data for the smoking-related variables. The study assistants who conducted the computer-assisted telephone interviews at follow-up were blinded to group allocation when assessing the primary and secondary outcome measures.

### Sample Size Calculation

Based on results of a study that tested the efficacy of telephone counseling for smoking cessation in high school students [8], we expected an 8% difference in 7-day point prevalence abstinence rates between the intervention and the control condition at 6-month follow-up assessment (25% vs 17%, respectively). To achieve a power of .80 with a significance level of .05 using a chi-square test (χ^2^), a sample size of n=406 in each study group was necessary. Because students were nested within school classes, we also needed to consider a potential design effect of 1.12 (average cluster size n=7; intracluster correlation coefficient: 0.02), which resulted in a required sample size of n=455 per study group.

### Intervention

#### Technological Background

The text messaging intervention, SMS-COACH, was fully automated and based on Internet technology using a Linux, Apache, MySQL, and PHP (LAMP) system. The program used in the present study was an extended and modified version of a previous version that had been tested successfully in pilot studies [16-18]. All incoming and outgoing text messages were automatically recorded. Incoming messages were analyzed immediately.

#### Theoretical Background

The program was primarily based on the HAPA [20]. This health behavior model suggests a distinction between motivation processes resulting in goal setting and volition processes leading to the actual health behavior. The approach combines 3 nonactive stages (precontemplation, contemplation, and preparation) and 2 active stages (action and maintenance). Within the initial 2 stages, outcome expectancies, risk perception, and perceived self-efficacy are important social-cognitive predictors to develop an intention to act. Within the subsequent intentional stage (preparation), planning processes are crucial to achieve the desired action. Once an action has been initiated, self-regulatory skills are important to maintain the healthy behavior. In addition to the HAPA, we used intervention elements derived from the Social Norms Approach [23] and implementation intentions, which are if-then plans that link situational cues with responses that are effective in attaining a desired outcome [24].

#### Intervention Elements

The intervention program consisted of (1) an online assessment of individual smoking behavior and attitudes toward smoking cessation, (2) a weekly SMS text message assessment of smoking-related target behaviors, (3) 2 weekly text messages tailored to the data of the online and the SMS text message assessments, and (4) an integrated quit day preparation and relapse-prevention program.

#### Online Baseline Assessment

In addition to the screening questions and the previously mentioned smoking-related variables that were assessed in both study groups at the baseline assessment, participants of the intervention group received online questions assessing (1) outcome expectancies of smoking cessation, (2) situations or circumstances in which craving for cigarettes usually occurs, (3) alternative strategies to handle these craving situations, and (4) costs per cigarette package.

#### Weekly Text Message Assessment

During the 3-month intervention period, participants in the intervention group received 1 text message per week to assess smoking-related target behavior. This question could be answered easily by typing a single letter or number, using the reply function of the mobile phone. The weekly SMS text message assessment question was sent at a fixed time point each week (6 pm on the weekday of study registration). The content of the question depended on the HAPA stage as well as on the number of the intervention week.

For all participants, the HAPA stage was assessed in even weeks by the question: “Have you recently smoked cigarettes?” with the following response options (1) “Yes, and I do not intend to quit” (precontemplation), (2) “Yes, but I am considering quitting” (contemplation), (3) “Yes, but I seriously intend to quit” (preparation), or (4) “No, I quit smoking” (action). This question assessed both smoking status and intention to quit over time. The responses to this question allowed the tailoring of the SMS text message feedback according to the current HAPA stage [25].

In odd weeks, we assessed the number of cigarettes smoked per day or week (depending on smoking status: daily/occasionally) in smokers in the preintentional stages (precontemplation and contemplation). We also assessed whether smokers in the intention or action stage applied the individually chosen strategies to cope with craving situations (eg, “Did you apply the following strategy recently? When I am at a party, I distract myself from smoking by dancing.”).

#### Individually Tailored Text Messages

At the first level, text messages were tailored to the HAPA stage. Persons in the preintentional stages received text messages addressing (1) the risks of smoking, (2) the monetary costs of smoking, (3) the social norms of smoking, (4) outcome expectancies, and (5) motivation to reduce the number of cigarettes smoked per day (daily smokers) or week (occasional smokers). Persons in the intentional stage received text messages that (1) motivated them to use social support for smoking cessation, (2) provided strategies to cope with craving situations, and (3) provided tips for preparing for smoking cessation (eg, reducing the number of cigarettes, identifying craving situations). Persons in the action stage received text messages (1) motivating them to reward themselves for staying abstinent, (2) providing strategies to cope with craving situations, and (3) motivating them to use social support for staying abstinent.

On the second level, the text messages were tailored according to the individual information provided at the baseline assessment as well as through the weekly SMS text message assessments. Examples of text messages are displayed in the study protocol of this trial [19] or in Multimedia Appendix 1.

#### Integrated Program for Quit Day Preparation and Relapse Prevention

Persons in the preparation and action stage had the possibility to additionally participate in an integrated program for quit day preparation and relapse prevention. Program participants in these stages were informed biweekly about this option. After entering a scheduled quit date, the program provided up to 2 daily text messages (weeks –1 to +1: 2 daily SMS text messages; weeks +2 and +3: 1 daily text message) to prepare for the quit day and to prevent relapse afterwards.

#### Number of Text Messages Sent to the Participants

Participants who did not use the integrated program for quit day preparation and relapse prevention received a total of 37 text messages (1 welcome message, 11 assessment messages, 24 tailored feedback messages, 1 goodbye message). Participants, who used the quit day preparation and relapse-prevention program for the whole period from 1 week before the scheduled quit date until 3 weeks afterwards, received an additional 42 text messages.

### Control Group

Study participants in the assessment-only control group did not receive any of the previously described intervention elements of the SMS-COACH program.

#### Baseline Measures

The screening assessment included the following demographic variables: gender, age, school education, and immigration background. Common Swiss levels of educational attainment were assessed: (1) none, (2) secondary school, (3) extended secondary school, and (4) technical or high school. We assessed the country of birth of both parents of the students to identify a potential immigrant background. Based on this information, participants were assigned to one of the following categories: (1) neither parent born outside Switzerland, (2) 1 parent born outside Switzerland, or (3) both parents born outside Switzerland.

The following health-related variables were assessed: physical activity and alcohol use. Self-reported moderate to vigorous physical activity was measured by a question derived from the Health Behavior in School-Aged Children (HBSC) study [26]: “Outside school, how many hours a week do you exercise or participate in sports that make you sweat or out of breath?” Alcohol consumption was assessed using the first 3 items about consumption of the Alcohol Use Disorder Identification Test (AUDIT-C), [27,28]. The AUDIT-C assesses drinking quantity, drinking frequency, and binge drinking. Based on recent recommendations [29], we used the gender-specific cut-off values for the AUDIT-C total score, ≥4 for men and ≥3 for women, to determine whether hazardous drinking was present.

Tobacco smoking was assessed using the question, “Are you currently smoking cigarettes or did you smoke in the past?” with the following response options: (1) I smoke cigarettes daily; (2) I smoke cigarettes occasionally, but not daily; (3) I smoked cigarettes in the past, but I do not smoke anymore; and (4) I have never smoked cigarettes or have smoked less than 100 cigarettes in my life. In occasional smokers, we additionally assessed the number of days they typically smoked per month and the total number of cigarettes smoked within the previous 7 days. In daily smokers and occasional smokers who smoked at least 4 cigarettes in the preceding month and at least 1 cigarette during the preceding week, we additionally assessed the following smoking-related variables: mean number of cigarettes smoked per day, stage of change according to the HAPA, and number of previous quit attempts.

In daily smokers, we assessed the number of cigarettes smoked on a typical day. In occasional smokers, we initially assessed the typical number of smoking days per month; subsequently, the number of cigarettes smoked on a typical smoking day was assessed. For occasional smokers, the number of cigarettes smoked per day was computed by multiplying the typical number of smoking days per month by the number of cigarettes smoked on a typical smoking day divided by 30. The stage of change based on the HAPA was assessed by the following question: “Have you recently smoked cigarettes?” with the following response options (1) “Yes, and I do not intend to quit” (precontemplation), (2) “Yes, but I am considering quitting” (contemplation), and (3) “Yes, but I seriously intend to quit” (preparation). Previous quit attempts were assessed by the question: “Have you ever made a serious attempt to quit smoking?” with the response options (1) no, (2) yes, once, and (3) yes, more than once. Furthermore, we assessed age at smoking onset by the question: “How old were you when you started smoking periodically?”

#### Program Participation and Program Use

To evaluate acceptance of the program, we analyzed log files of the SMS text message system in which the number and content of incoming and outgoing text messages were recorded. The number of responses to the weekly SMS text message assessments and the number of program participants who unsubscribed from the program (program attrition) were examined. At follow-up, we also assessed usage of the SMS text messages by asking the participants whether they (1) read the SMS text message feedback messages thoroughly, (2) took only a short look at the feedback messages, or (3) did not read the feedback messages.

#### Follow-Up Measures

Computer-assisted telephone interviews were conducted at the 6-month follow-up assessment by trained interviewers. The following outcome variables were assessed during this interview: (1) smoking status, (2) 7-day smoking abstinence, (3) 4-week smoking abstinence, (4) mean number of cigarettes smoked per day, (5) stage of change according to the HAPA, and (6) quit attempts within the past 6 months preceding the follow-up. The main outcome criterion was 7-day point prevalence smoking abstinence.

For assessment of smoking status, the participants could indicate whether they smoked (1) daily, (2) occasionally, or (3) do not smoke anymore. Furthermore, 7-day point prevalence smoking abstinence (ie, not having smoked a puff within the past 7 days preceding the follow-up [23]), and 4-week point prevalence smoking abstinence were assessed. Among daily smokers, we assessed the number of cigarettes smoked on a typical day. Among occasional smokers, we initially assessed the typical number of smoking days per month and subsequently the number of cigarettes smoked on a typical smoking day. For occasional smokers, the number of cigarettes smoked per day was computed by multiplying the typical number of smoking days per month by the number of cigarettes smoked on a typical smoking day divided by 30. In participants who indicated that they did not smoke anymore, the value for the number of cigarettes smoked per day was set to zero.

The HAPA stage was assessed by a similar question as at baseline. Participants indicating that they did not smoke anymore were assigned to the action stage. Quit attempts within the previous 6 months were assessed by the yes/no question: “Have you made a serious attempt to quit smoking within the previous 6 months?” For participants who indicated that they did not smoke anymore, a serious quit attempt was assumed.

#### Data Analysis

The data were analyzed using STATA software, version 10. To test for baseline equivalence of intervention and control individuals, chi-square tests for categorical variables and *t* tests for continuous variables were used. For the attrition analysis (study participants lost to follow-up), we also used chi-square tests for categorical variables and *t* tests for continuous variables. Baseline equivalence and lack of attrition bias were assumed for tests with *P*>.10.

We used regression models to verify the efficacy of the intervention on the different outcome measures. Logistic regression models were applied for the binary outcome variables (7-day and 4-week point prevalence smoking abstinence), negative binomial regression models were applied for the count data (number of cigarettes smoked per day), ordinal logistic regression models were used for ordinal data (stage of change), and multinomial logistic regression models were used for categorical outcomes (smoking status). To control for baseline differences, we additionally added the respective baseline variables as covariates to the regression models.

We conducted both complete-case analyses (CCA) considering all study participants with available follow-up data, and intention-to-treat (ITT) analyses. For the ITT analyses, we applied the multiple imputations procedure (MICE) of STATA, which imputed missing follow-up data by using all available baseline variables (demographic, health- and smoking-related variables). We created 30 imputed datasets. Given the clustered nature of the data (students within school classes), we computed robust variance estimators for all regression models using the svy command of STATA.

Because of significant baseline differences between the study groups, particularly in the percentage of occasional and daily smokers, and significant interaction effects of study condition × smoking status for the number of cigarettes smoked per day (*P*=.01) and quit attempts within the previous 6 months (*P*=.02) outcomes, we additionally conducted outcome analyses separately for occasional and daily smokers.

## Results

### Study Participation

**Figure 1:**
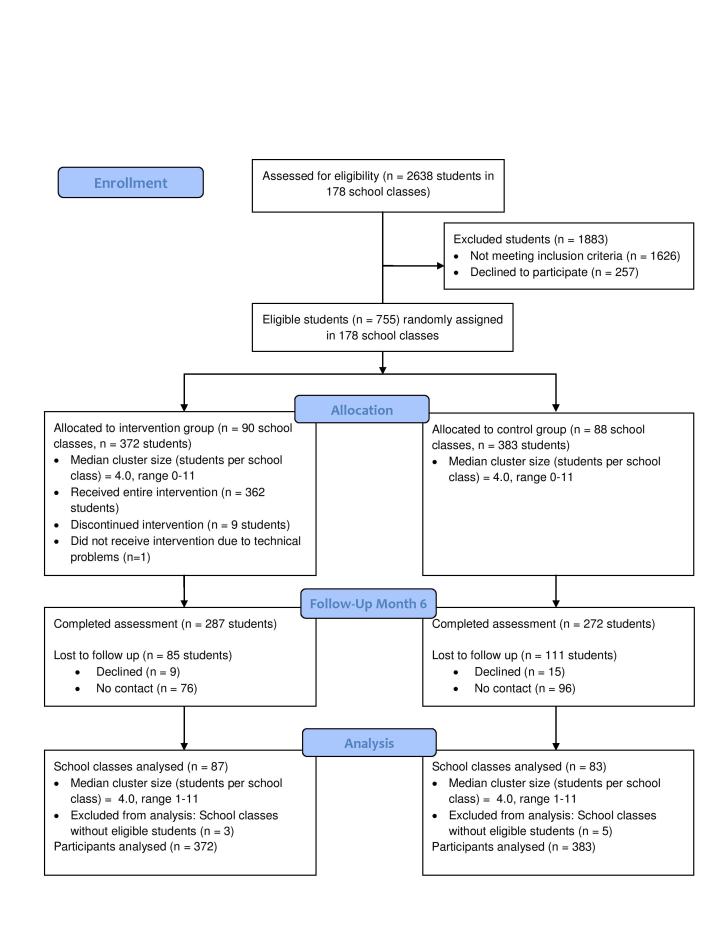
Flowchart of study participants.

Figure 1 presents a flowchart of the study participants. At the time of the online screening assessment in 178 school classes, a total of 2657 students were present. Among them, 2638 (99.3%) agreed to participate. Of these, 1012 persons met the inclusion criteria for study participation and 755 persons (74.6%) participated in the study. Ninety classes consisting of 372 students were randomly assigned to the intervention group and 88 classes consisting of 383 students were assigned to the control group. Follow-up assessments were completed in 287 (77.2%) study participants in the intervention group and 272 (71.0%) study participants in the control group.

### Sample Characteristics

Baseline characteristics for the study sample are shown in Table 1.

Baseline differences between intervention and control group participants were found for the following variables: gender (χ^2^_1_=3.1, *P*=.08), hazardous drinking (χ^2^_1_=4.8, *P*=.03), smoking status (χ^2^_1_=13.3, *P*<.001), number of cigarettes smoked per day (*t*_753_=3.6, *P*<.001), and age of onset of smoking (*t*_753_=–2.8, *P*=.005).

We conducted ancillary separate analyses for occasional and daily smokers, and then we checked for baseline differences within these subgroups. Within the sample of occasional smokers, the following baseline differences between intervention and control group participants were found: (1) a higher percentage of male participants in the intervention group (χ^2^_1_=4.3, *P*=.04), and (2) a higher number of cigarettes smoked per day in the intervention group (*t*_176_=–1.7, *P*=.09). Within the sample of daily smokers, the following baseline differences between intervention and control group participants were found: (1) a lower percentage of hazardous drinking in the intervention group (χ^2^_1_=5.3, *P*=.02), (2) lower cigarette consumption in the intervention group (*t*_575_=1.9, *P*=.06), and (3) a higher age of onset of smoking in the intervention group (*t*_575_=–1.8, *P*=.07).

The attrition analysis revealed that individuals lost to follow-up were more likely to be daily smokers (81.1% vs 74.8%; χ^2^_1_=3.2, *P*=.07) and smoked a higher number of cigarettes per day (11.5 vs 10.3; *t*_753_=2.0, *P*=.048).

**Table 1:** Demographics and health- and smoking-related baseline characteristics of the study sample.

Variable		Intervention n=372	Control n=383	Total N=755	*P* ^a^
**Gender, n (%)**					
	Male	191 (51.3)	172 (44.9)	363 (48.1)	.08^b^
	Female	181 (48.7)	211 (55.1)	392 (51.9)	
Age, mean (SD)		18.2 (2.4)	18.3 (2.2)	18.2 (2.3)	.56^c^
**Immigration background, n (%)**					.89^b^
	No immigration background	196 (52.7)	206 (53.8)	402 (53.2)	
	One parent born outside Switzerland	75 (20.2)	79 (20.6)	154 (20.4)	
	Both parents born outside Switzerland	101 (27.2)	98 (25.6)	199 (26.4)	
**Education, n (%)**					.68^b^
	None	14 (3.8)	11 (2.9)	25 (3.3)	
	Secondary school	290 (78.0)	301 (78.6)	591 (78.3)	
	Extended secondary school	59 (15.9)	56 (14.6)	115 (15.2)	
	Technical or high school	9 (2.4)	15 (3.9)	24 (3.2)	
**Hazardous drinking, n (%)**					.03^b^
	No	76 (20.4)	55 (14.4)	131 (17.4)	
	Yes	296 (79.6)	328 (85.6)	624 (82.6)	
Hours of extracurricular moderate to vigorous physical activity per week, mean (SD)		4.0 (4.6)	3.7 (5.0)	3.8 (4.8)	.37^c^
**Tobacco smoking status, n (%)**					<.001^b^
	Occasional smoker	109 (29.3)	69 (18.0)	178 (23.6)	
	Daily smoker	263 (70.7)	314 (82.0)	577 (76.4)	
**Stage of change, n (%)**					.11^b^
	No intention to quit	86 (23.1)	112 (29.2)	198 (26.2)	
	Considering quitting	214 (57.5)	211 (55.1)	425 (56.3)	
	Serious intention to quit	72 (19.4)	60 (15.7)	132 (17.5)	
Number of cigarettes smoked per day, mean (SD)		9.6 (7.2)	11.6 (7.9)	10.6 (7.6)	<.001^c^
Age of onset of smoking, mean (SD)		15.1 (1.6)	14.8 (1.7)	15.0 (1.6)	.005^c^
**Previous quit attempts, n (%)**					.32^b^
	0	148 (39.8)	141 (36.8)	289 (38.3)	
	1	153 (41.1)	178 (46.5)	331 (43.8)	
	2 or more	71 (19.1)	64 (16.7)	135 (17.9)	

^a^ *P* values for the comparison of intervention and control group participants.

^b^χ^2^ test.

^c^ *t* test.

### Program Attrition and Program Use

During the program, which lasted for 3 months, 9 (2.4%) of the 372 participants in the intervention group unsubscribed from the program.

The mean number of replies to the weekly SMS text message assessments was 6.5 (SD 3.7). No reply was sent by 34 participants (9.1%), and all 11 replies were sent by 55 participants (14.8%).

Out of the 287 participants with valid follow-up data, 271 (94.4%) indicated that they regularly read the SMS text messages. Of these, 204 (75.3%) indicated that they read the SMS text messages thoroughly, whereas 67 participants (24.7%) reported that they took a short look at the feedback messages.

### Program Efficacy

#### Smoking Abstinence

Table 2 presents 7-day and 4-week point prevalence smoking abstinence rates at follow-up for both study groups based on complete case data. Using CCA and ITT, the logistic regression analyses controlling for differences in baseline characteristics did not reveal any differences in 7-day or 4-week smoking abstinence rates at follow-up between the study groups for the total sample, the subgroup of occasional smokers and the subgroup of daily smokers.

#### Cigarette Consumption

Table 3 presents the mean number of cigarettes smoked per day at follow-up for both study groups based on complete case data. Both CCA and ITT revealed lower cigarette consumption in the intervention group than in the control group. Within baseline occasional smokers and baseline daily smokers, both CCA and ITT revealed lower cigarette consumption in the intervention group than in the control group.

#### Stage of Change

Table 4 presents the stage of change at follow-up for participants in both study groups. Using CCA and ITT, the regression models did not reveal differences in stages of change between the study groups for the total sample, the subgroup of occasional smokers, and the subgroup of daily smokers.

#### Quit Attempts

Based on complete case data of the total sample, 98 (36.3%) of 270 participants in the control group and 125 (43.7%) of 286 participants in the intervention group indicated that they made a quit attempt within the 6 months preceding follow-up (CCA: OR 1.17, 95% CI 0.81-1.71, *P*=.40; ITT: OR 1.18, 95% CI 0.81-1.72, *P*=.38). In baseline occasional smokers, 12 (43.1%) of 51 participants in the control group and 62 (68.9%) of 90 participants in the intervention group indicated a quit attempt (CCA: OR 2.79, 95% CI 1.36-5.73, *P*=.006; ITT: OR 2.48, 95% CI 1.24-4.93, *P*=.01). Using the subgroup of baseline daily smokers, 76 (34.7%) of 219 participants in the control group and 63 (32.1%) of 196 participants in the intervention group indicated a quit attempt (CCA: OR 0.87, 95% CI 0.55-1.37, *P*=.54; ITT: 0.95, 95% CI 0.62-1.46, *P*=.82).

**Table 2:** Point prevalence smoking abstinence rates at follow-up (complete-case data) and results of logistic regression analyses comparing abstinence rates in the study groups using complete-case analyses (CCA) and intention-to-treat analyses (ITT).

Sample		Control n (%)	Intervention n (%)	OR (95% CI)		*P*	
				CCA	ITT	CCA	ITT
**Total sample** ^a^							
	7-day abstinence	26 (9.6)	36 (12.5)	1.02 (0.60-1.76)	1.03 (0.59-1.79)	.93	.92
	4-week abstinence	15 (5.5)	18 (6.3)	0.87 (0.45-1.71)	0.97 (0.50-1.90)	.69	.92
**Baseline occasional smokers** ^b^							
	7-day abstinence	10 (19.6)	25 (27.8)	1.64 (0.65-4.10)	1.56 (0.65-3.75)	.29	.32
	4-week abstinence	4 (7.8)	13 (14.4)	1.99 (0.58-6.80)	2.06 (0.63-6.78)	.27	.23
**Baseline daily smokers** ^c^							
	7-day abstinence	16 (7.2)	11 (5.6)	0.83 (0.35-1.92)	0.81 (0.36-1.81)	.65	.61
	4-week abstinence	11 (5.0)	5 (2.5)	0.48 (0.13-1.80)	0.55 (0.17-1.77)	.27	.32

^a^Based on 272 participants in the control group and 287 in the intervention group.

^b^Based on 51 participants in the control group and 90 in the intervention group.

^c^Based on 221 participants in the control group and 197 in the intervention group.

**Table 3:** Mean number of cigarettes smoked per day at follow-up (complete case data) and results of logistic regression analyses comparing cigarette consumption in the study groups using complete-case analyses (CCA) and intention-to-treat analyses (ITT).

Sample	Control mean (SD)	Intervention mean (SD)	*t* (*df*)		*P*	
			CCA	ITT	CCA	ITT
Total sample	10.0 (7.9)	7.5 (7.2)	–2.80 (164)	–3.18 (43.1)	.006	.002
Baseline occasional smokers	2.7 (3.2)	1.7 (2.4)	–2.32 (89)	–2.41 (125.9)	.02	.02
Baseline daily smokers	11.7 (7.7)	10.2 (7.1)	–2.22 (151)	–2.53 (37.5)	.03	.01

**Table 4:** Stage of change at follow-up based on complete case data and results of ordinal regression analyses comparing stage of change between the study groups using complete-case analyses (CCA) and intention-to-treat analyses (ITT).

Sample		Control n (%)	Intervention n (%)	*t* (*df*)		*P*	
				CCA	ITT	CCA	ITT
**Total sample** ^a^				0.33 (164)	0.23 (78.7)	.74	.82
	Precontemplation	72 (26.7)	65 (22.7)				
	Contemplation	133 (49.3)	134 (46.9)				
	Preparation	29 (10.7)	32 (11.2)				
	Action	36 (13.3)	55 (19.2)				
**Baseline occasional smokers** ^b^				1.60 (89)	1.33 (78.9)	.11	.18
	Precontemplation	8 (15.7)	5 (5.6)				
	Contemplation	22 (43.1)	38 (42.2)				
	Preparation	6 (11.8)	6 (6.7)				
	Action	15 (29.4)	41 (45.6)				
**Baseline daily smokers** ^c^				-0.24 (151)	-0.40 (89.4)	.81	.69
	Precontemplation	64 (29.2)	60 (30.6)				
	Contemplation	111 (50.7)	96 (49.0)				
	Preparation	23 (10.5)	26 (13.3)				
	Action	21 (9.6)	14 (7.1)				

^a^Based on 270 participants in the control group and 286 in the intervention group.

^b^Based on 51 participants in the control group and 90 participants in the intervention group.

^c^Based on 219 participants in the control group and 196 participants in the intervention group.

## Discussion

The study aimed to test the efficacy of an SMS text message–based intervention for smoking cessation in a sample of proactively recruited vocational school students with different motivation to quit. The study revealed 4 main findings: (1) a large percentage of smoking students participated in the program, (2) program attrition was low, (3) program participation resulted in lower cigarette consumption, but (4) no short-term effect of the intervention on smoking abstinence rates was found.

The proactive invitation for program participation in combination with the offer of a low-threshold intervention using SMS text messages allowed us to reach 3 of 4 smoking students (75%) for participation in the SMS-COACH program. Taking into account that 83% of the program participants were in the precontemplation or contemplation stage at baseline (ie, indicated no serious intention to quit), this high participation rate is of special relevance. Other school-based smoking cessation interventions conducted in German-speaking countries showed much lower participation rates of 37% [30] and 19% [11]. In-line with other recently developed smoking cessation approaches in adolescents [7,8], our results underscore the importance of proactive recruitment strategies and low-threshold interventions to attain a high participation rate. The flexibility of SMS text messaging to send and receive messages at any time, place, or setting, as well as the possibility to receive individually tailored information, might be responsible for the high use and retention rates identified in this study. Nearly all program participants (98%) stayed logged in until the end of the 3-month program. The SMS text messages were read by almost all program participants (94%) and 9 of 10 program participants (91%) replied to the SMS text message assessments.

The finding that the intervention program resulted in lower cigarette consumption indicates that the intervention might promote smoking abstinence. The number of cigarettes smoked per day, which is closely related to nicotine dependence [31], has proved to be among the best predictors of smoking cessation in both adolescents and adults [32-34]. However, the main study outcome was 7-day point prevalence smoking abstinence assessed at the 6-month follow-up. This abstinence rate was 12.5% in the intervention group and 9.6% in the control group. After controlling for baseline differences, no significant intervention effect was found for this criterion. The separate subgroup analyses for daily and occasional smokers also did not reveal an intervention effect on smoking abstinence. One explanation might be the short-term follow-up assessment, which was conducted 3 months after the end of the intervention. In motivational interventions addressing smokers irrespective of their intention to quit, the effects on smoking abstinence rates typically increase gradually [32,35] and might become statistically significant at later follow-up assessments.

The subgroup analyses revealed positive intervention effects for both subgroups on cigarette consumption. Furthermore, occasional smokers in the intervention group made more serious attempts to quit smoking. Quit attempts are significant predictors of smoking cessation [32,36,37].

Several limitations must be noted. First, smoking status was assessed by self-report and was not biochemically verified. However, we expect that a potential overreporting of smoking abstinence would be independent of the study condition. Furthermore, based on recommendations by the Society for Research on Nicotine and Tobacco, there are circumstances under which the added precision gained by biological validation is offset in such a way that its use is not required and may not be desirable [38]. Examples include population-based studies with low demands on smokers to quit (eg, interventions with limited face-to-face contact and studies in which the optimal data collection methods are through mail, telephone, or Internet). A second limitation is that we only investigated the short-term effects of the program. Longer follow-up assessments might provide different results. However, both of these limitations resulted in a lower expenditure of time for the study participants and a greater proximity to prevention practice. Therefore, they allowed a better estimation of the participation rate in the program that might be expected under routine intervention conditions. Further study limitations are the lack of statistical power, particularly for the subgroup analyses, and an attrition bias. Based on a higher percentage of daily smokers and higher cigarette consumption in individuals lost to follow-up as well as a higher percentage of persons lost to follow-up in the control group than in the intervention group, this attrition bias might have resulted in conservative estimations of intervention effects in the complete-case analyses.

The study demonstrates the potential of a text messaging–based intervention to reach a high proportion of young smokers with predominantly lower educational levels. The intervention resulted in statistically significant lower cigarette consumption in the total sample, the subgroup of occasional smokers, and the subgroup of daily smokers. Furthermore, it resulted in statistically significant more quit attempts in the subgroup of occasional smokers. No short-term effects were found according to the proportion of participants who had quit.

Both the baseline assessment and the registration for the SMS text message program are possible from every computer with Internet access and only take approximately 10 minutes. Therefore, the program could be easily implemented within school classes with low personnel expenses.

## Multimedia Appendix 1

Intervention components of the SMS program.

## Multimedia Appendix 2

CONSORT-EHEALTH checklist V1.6.2 [39].

## Abbreviations

AUDIT-C: Alcohol Use Disorder Identification Test (consumption questions)
CCA: complete-case analyses
HAPA: Health Action Process Approach
HBSC: Health Behavior in School-Aged Children
ITT: intention-to-treat
OR: odds ratio
SMS: short message service
