# Supplementary material for: Efficacy of a Text Message-Based Smoking Cessation Intervention for Young People: A Cluster Randomized Controlled Trial
Source: J Med Internet Res. 2013 Aug 16;15(8):e171. doi: 10.2196/jmir.2636 (PMC3757913; doi:10.2196/jmir.2636)
Supplement: Supplementary file 1 [file jmir_v15i8e171_app1.pptx]

## Slide 1
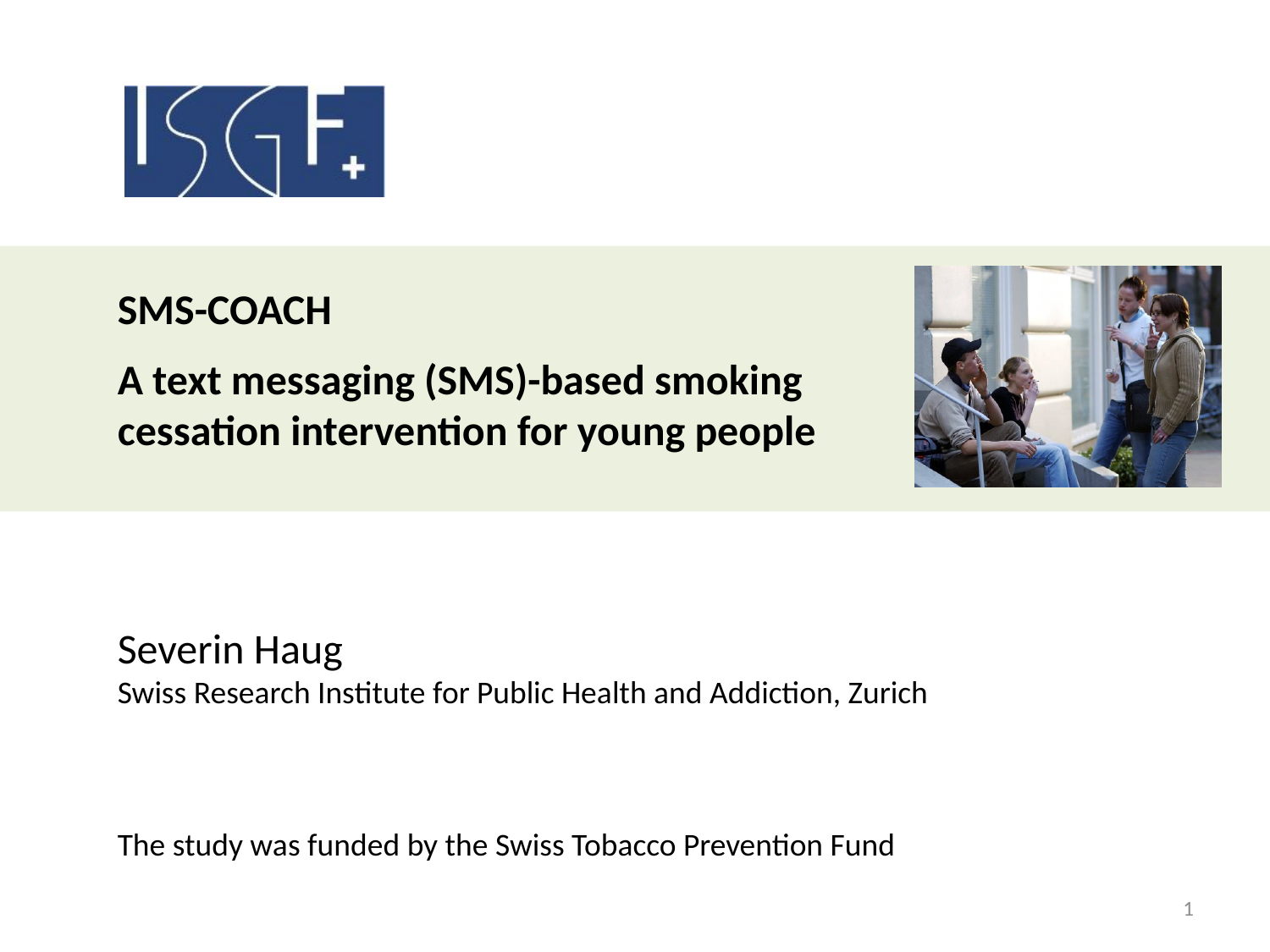

SMS-COACH
A text messaging (SMS)-based smoking
cessation intervention for young people
Severin Haug
Swiss Research Institute for Public Health and Addiction, Zurich
The study was funded by the Swiss Tobacco Prevention Fund
1

## Slide 2
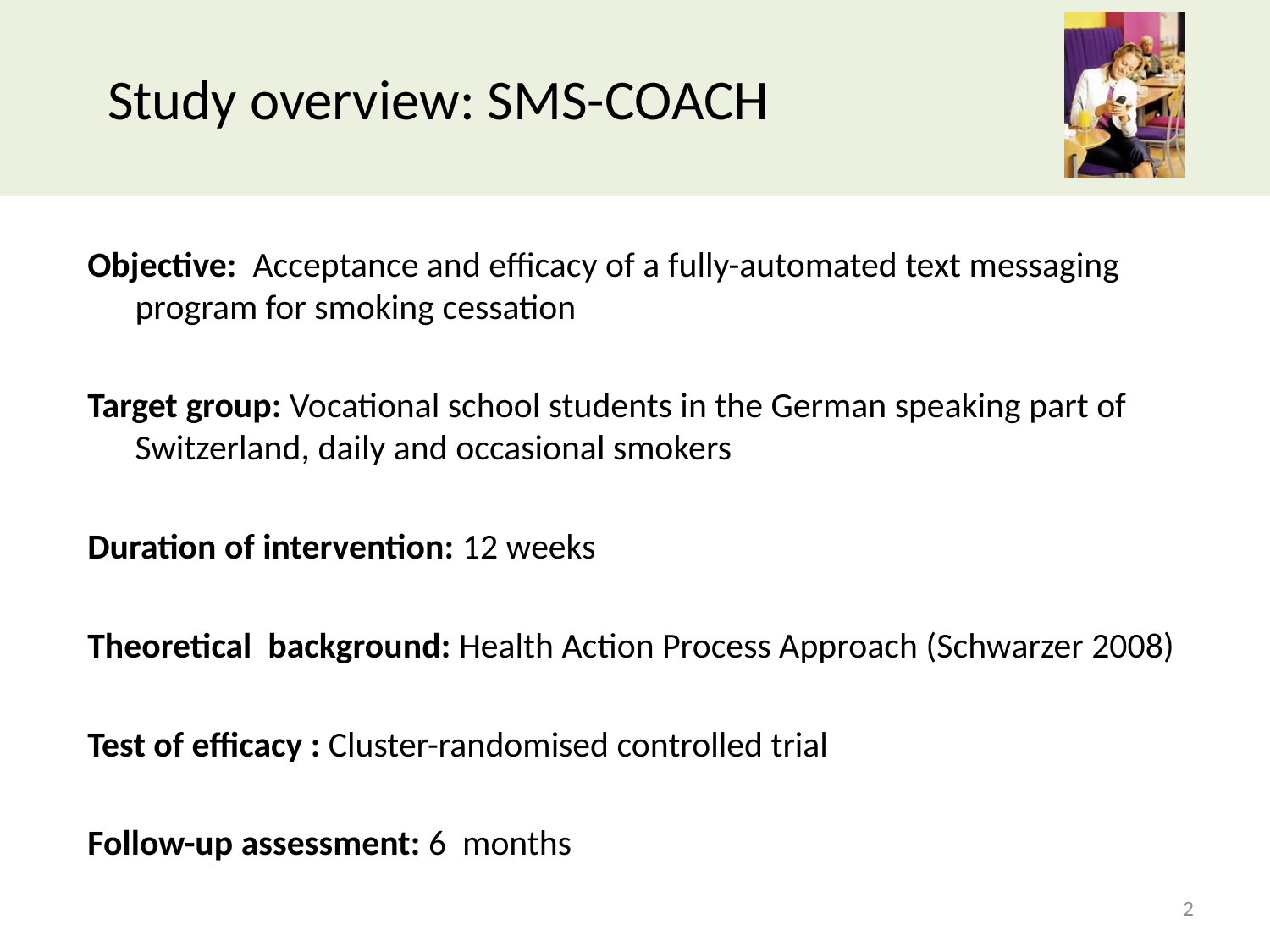

Study overview: SMS-COACH
Objective: Acceptance and efficacy of a fully-automated text messaging program for smoking cessation
Target group: Vocational school students in the German speaking part of Switzerland, daily and occasional smokers
Duration of intervention: 12 weeks
Theoretical background: Health Action Process Approach (Schwarzer 2008)
Test of efficacy : Cluster-randomised controlled trial
Follow-up assessment: 6 months
2

## Slide 3
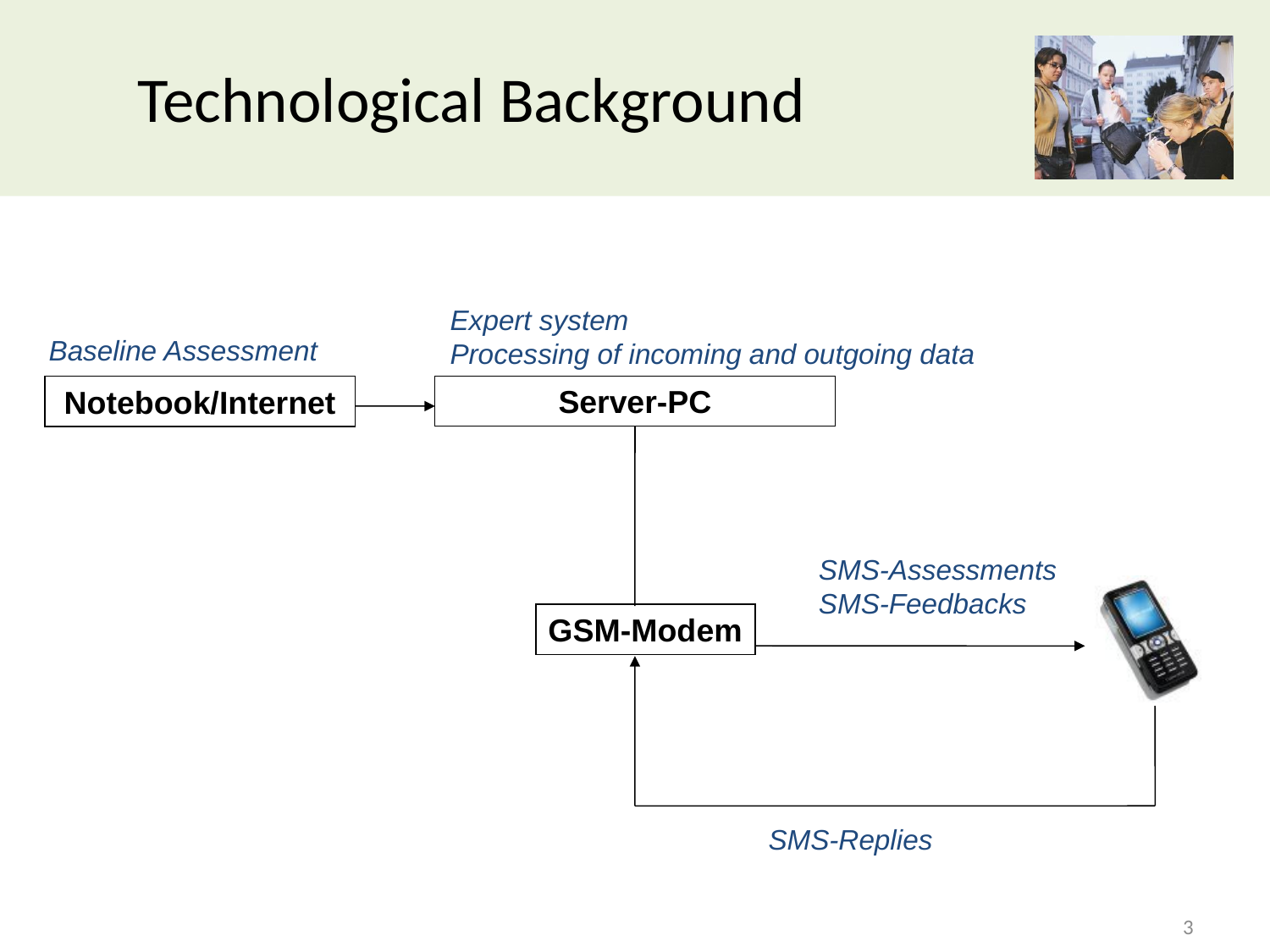

Technological Background
Expert system
Processing of incoming and outgoing data
Baseline Assessment
Server-PC
Notebook/Internet
SMS-Assessments
SMS-Feedbacks
GSM-Modem
SMS-Replies
3

## Slide 4
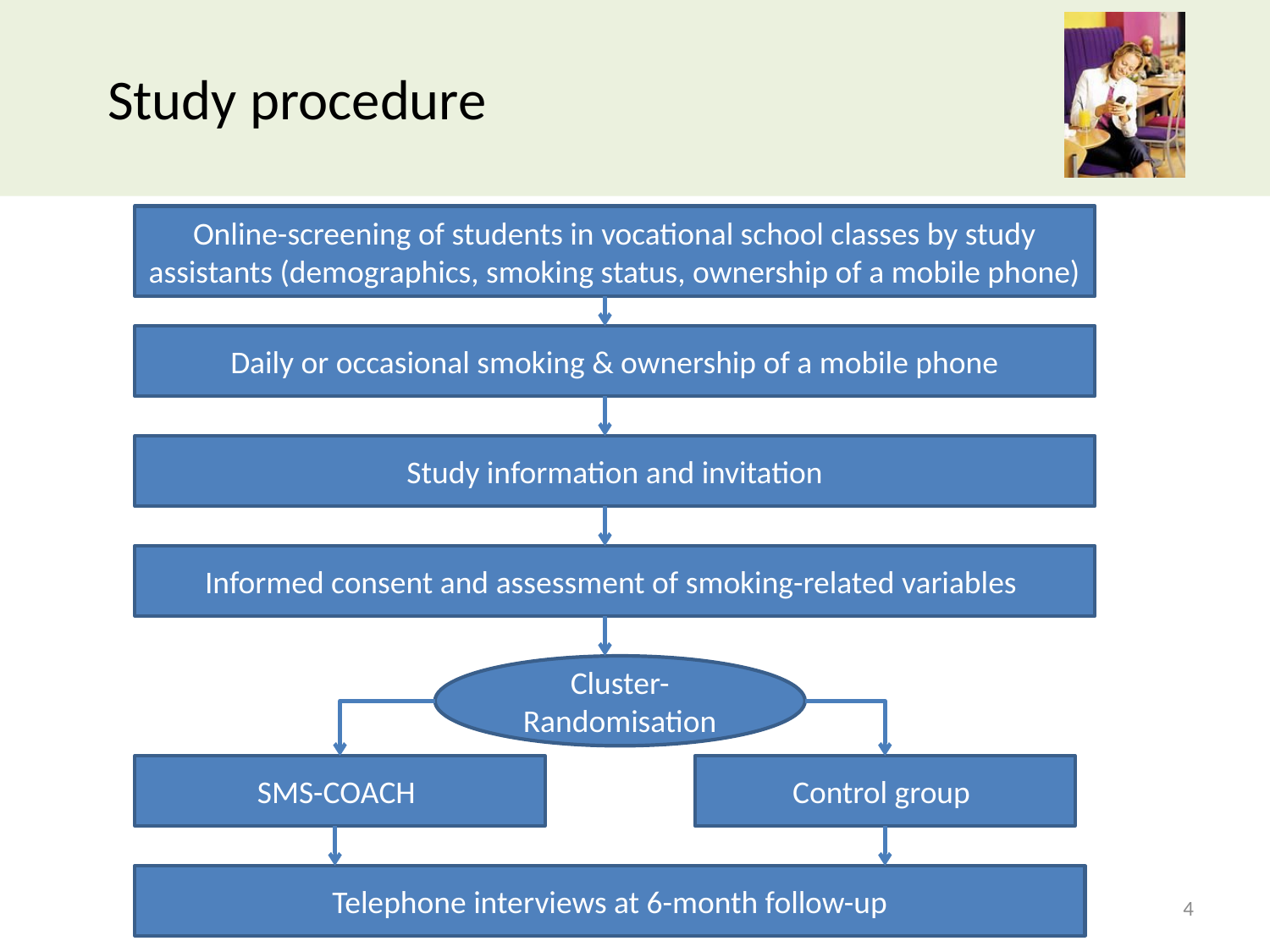

Study procedure
Online-screening of students in vocational school classes by study assistants (demographics, smoking status, ownership of a mobile phone)
Daily or occasional smoking & ownership of a mobile phone
Study information and invitation
Informed consent and assessment of smoking-related variables
Cluster-Randomisation
SMS-COACH
Control group
Telephone interviews at 6-month follow-up
4

## Slide 5
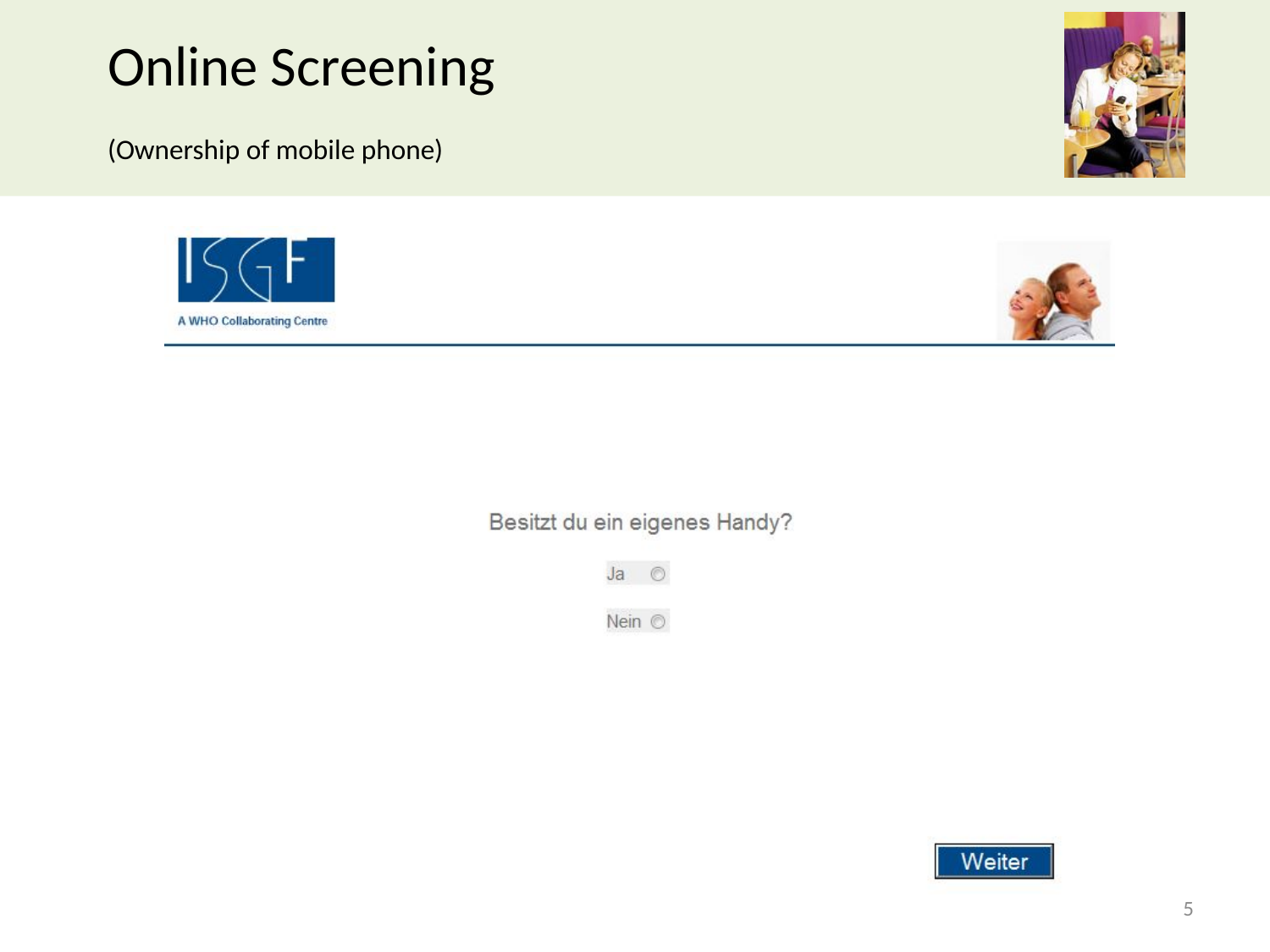

Online Screening
(Ownership of mobile phone)
5

## Slide 6
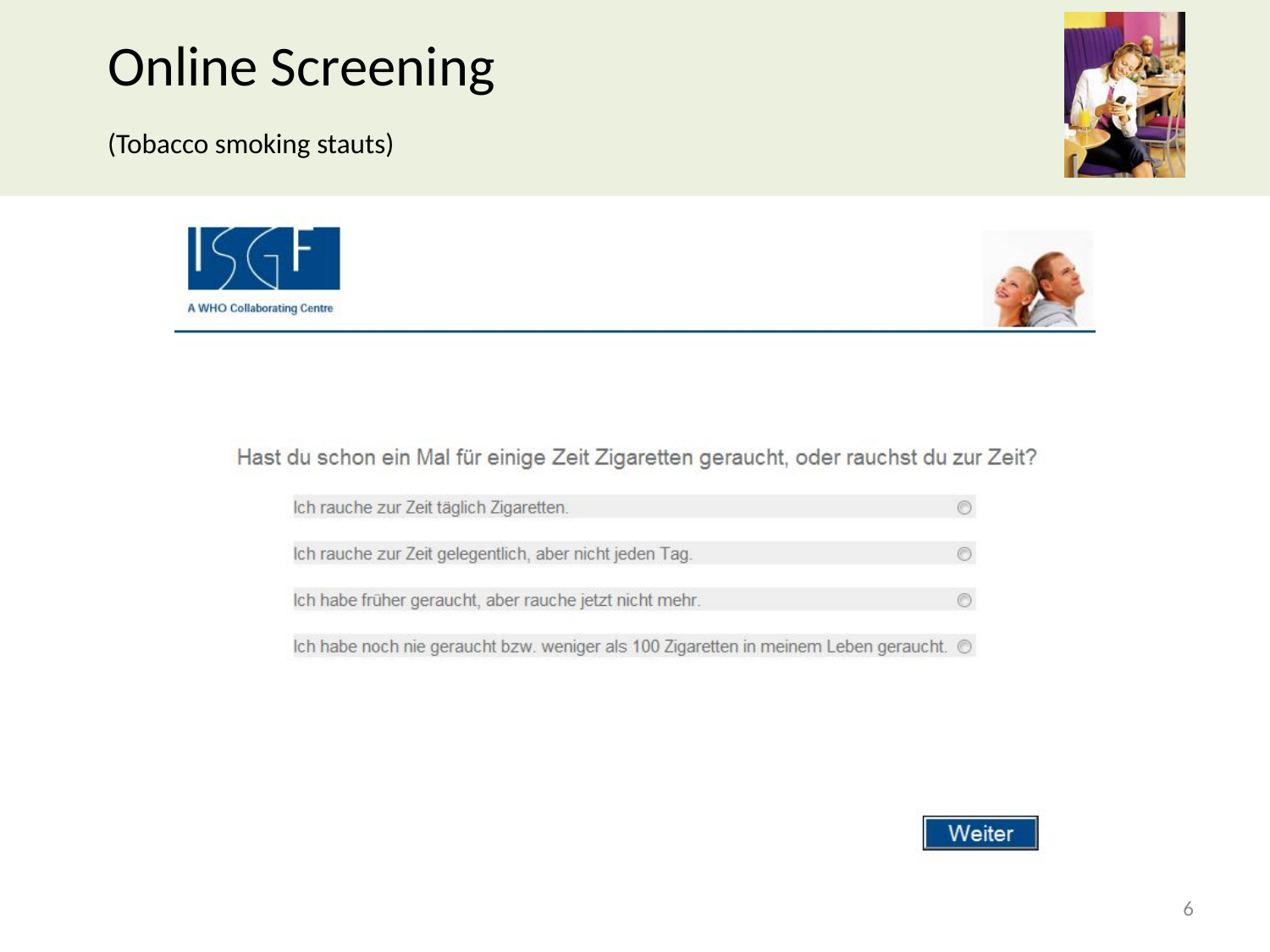

Online Screening
(Tobacco smoking stauts)
6

## Slide 7
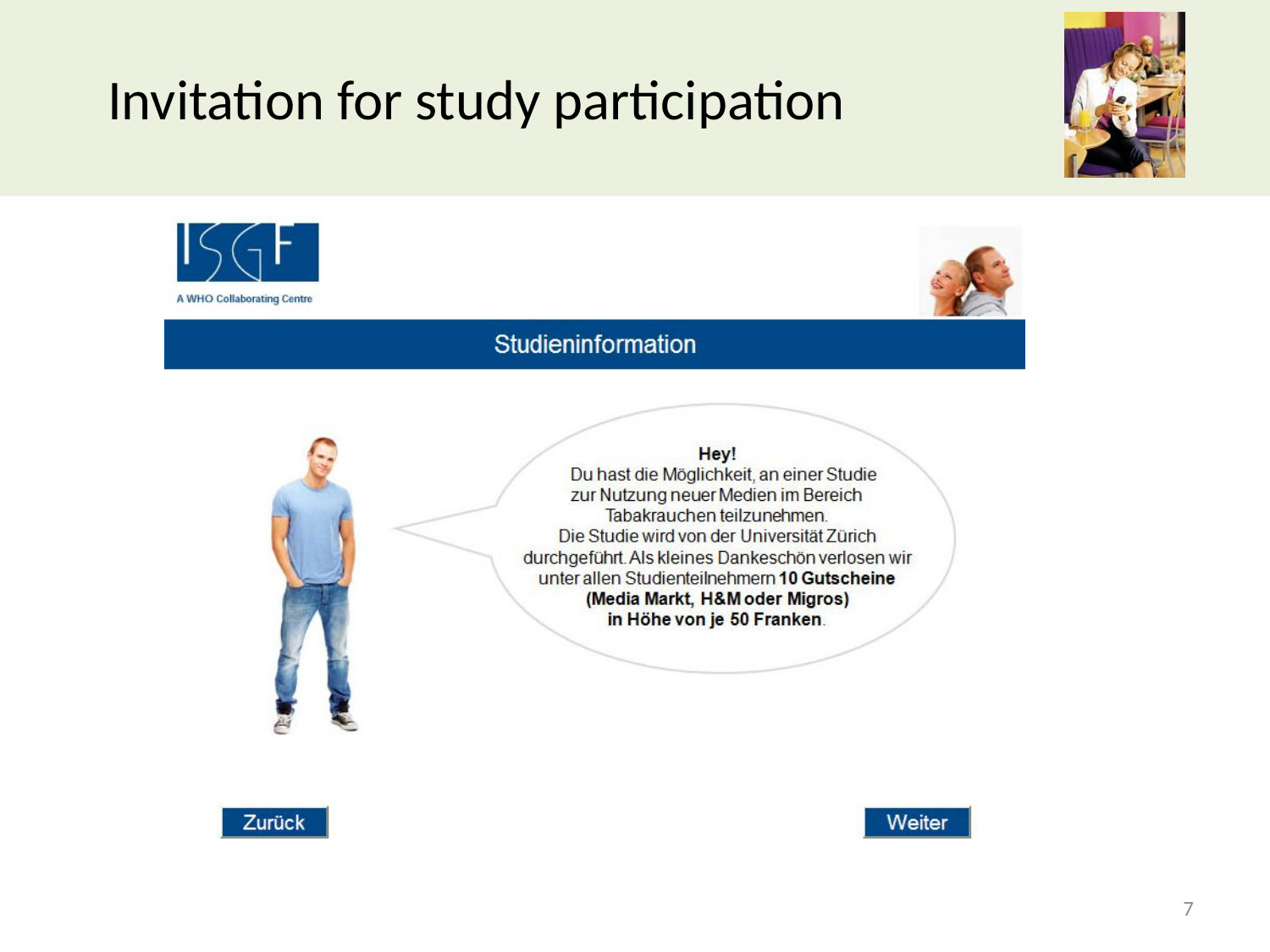

Invitation for study participation
7

## Slide 8
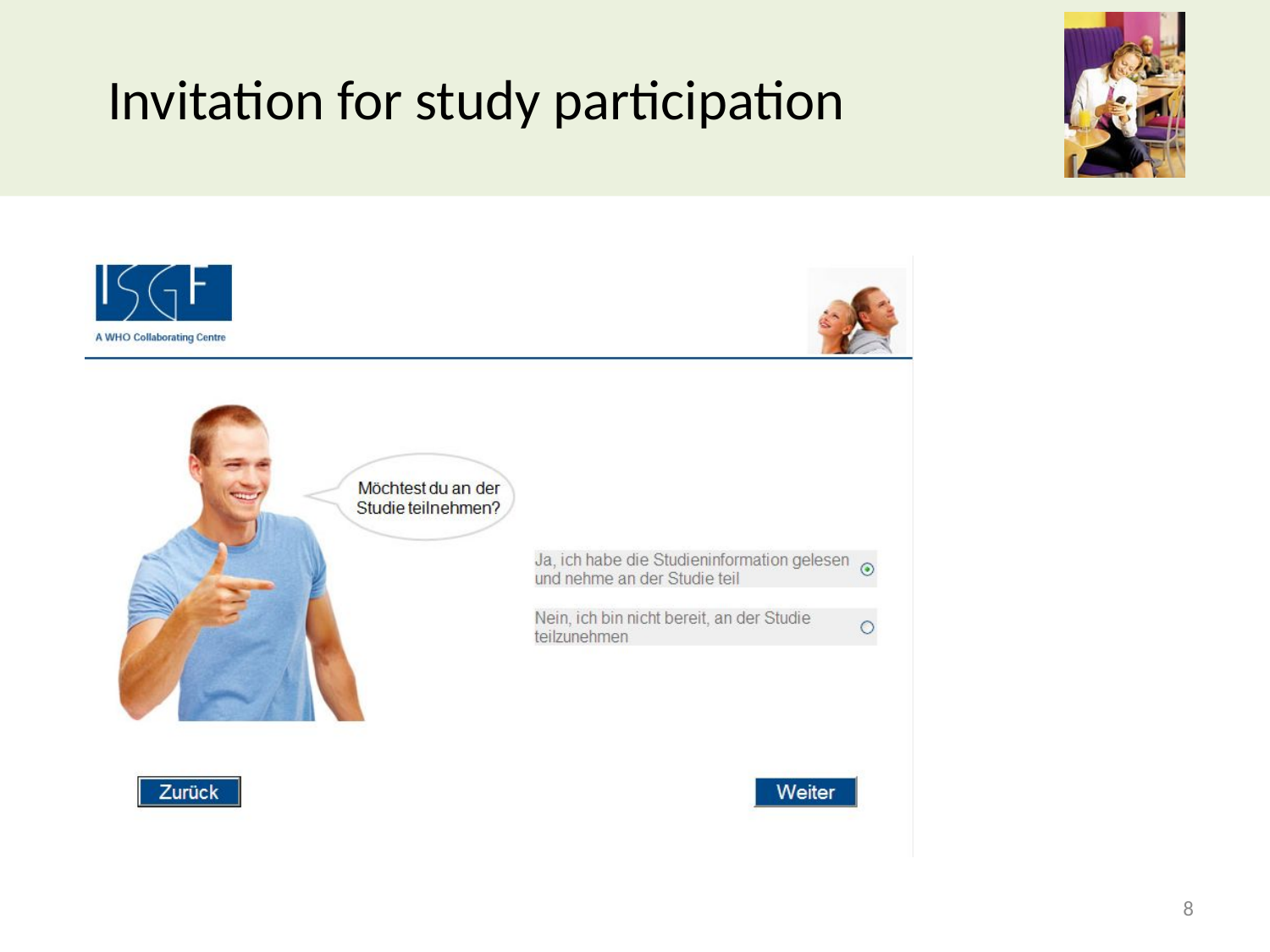

Invitation for study participation
8

## Slide 9
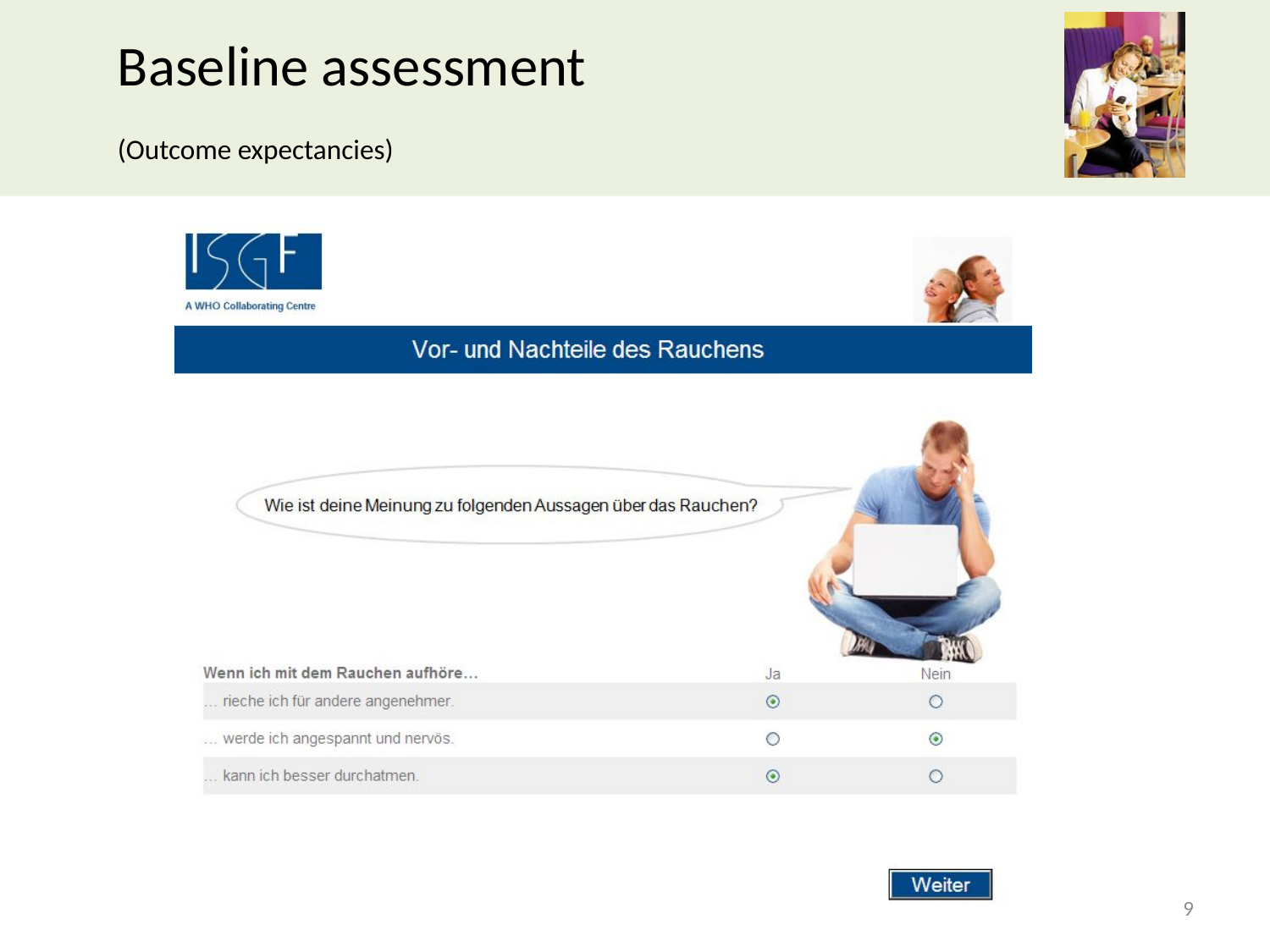

Baseline assessment
(Outcome expectancies)
9

## Slide 10
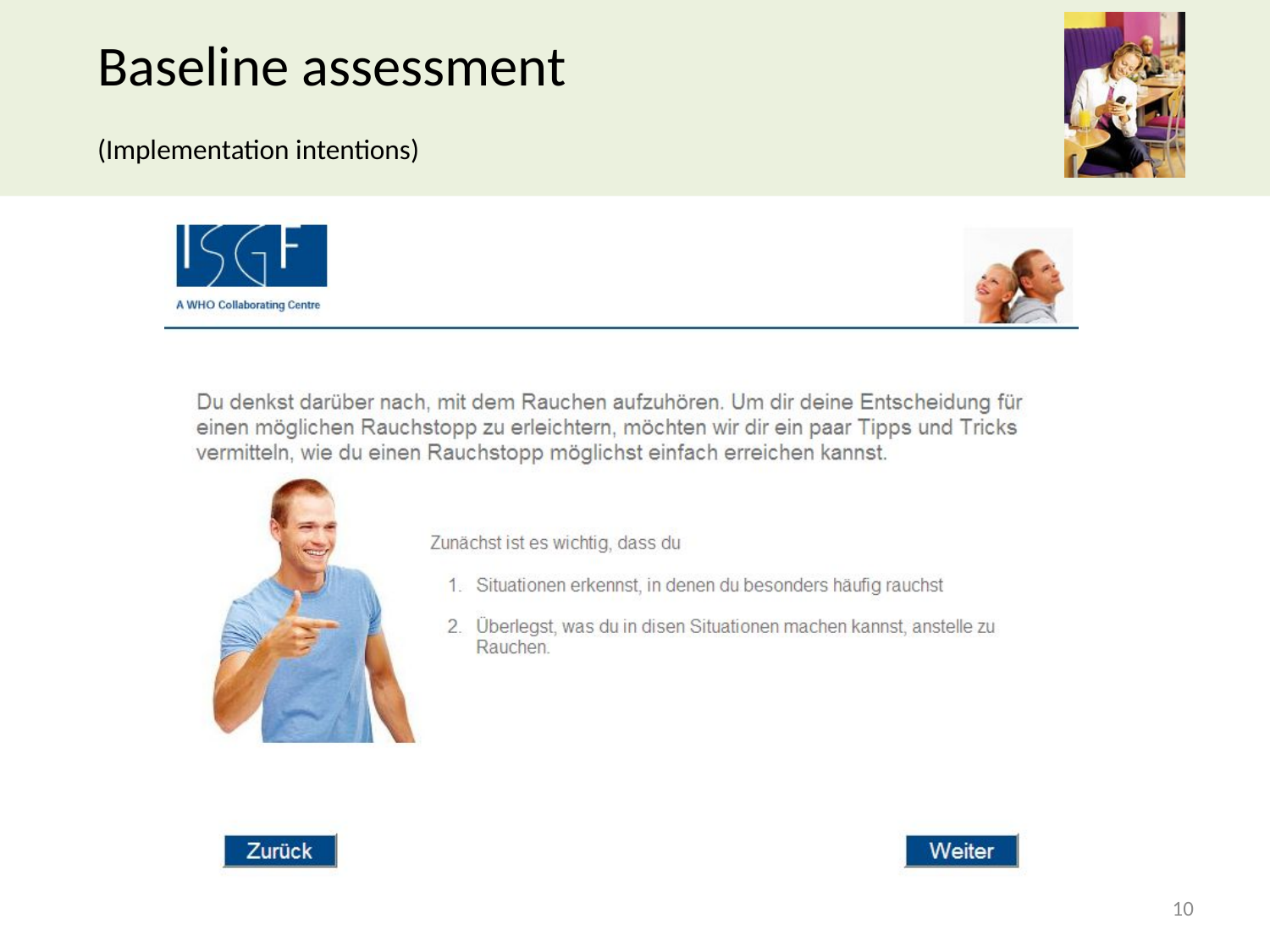

Baseline assessment
(Implementation intentions)
10

## Slide 11
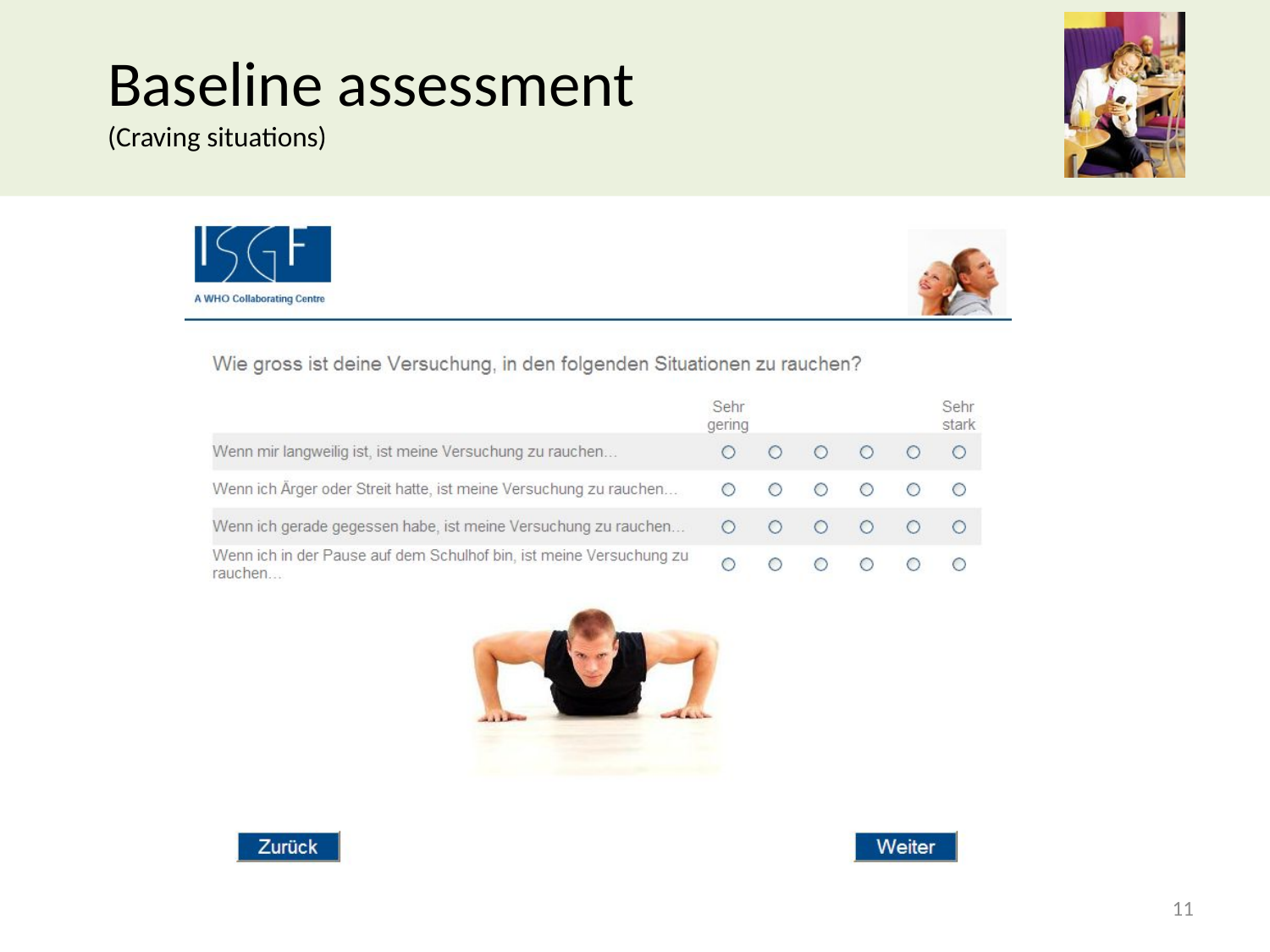

Baseline assessment
(Craving situations)
11

## Slide 12
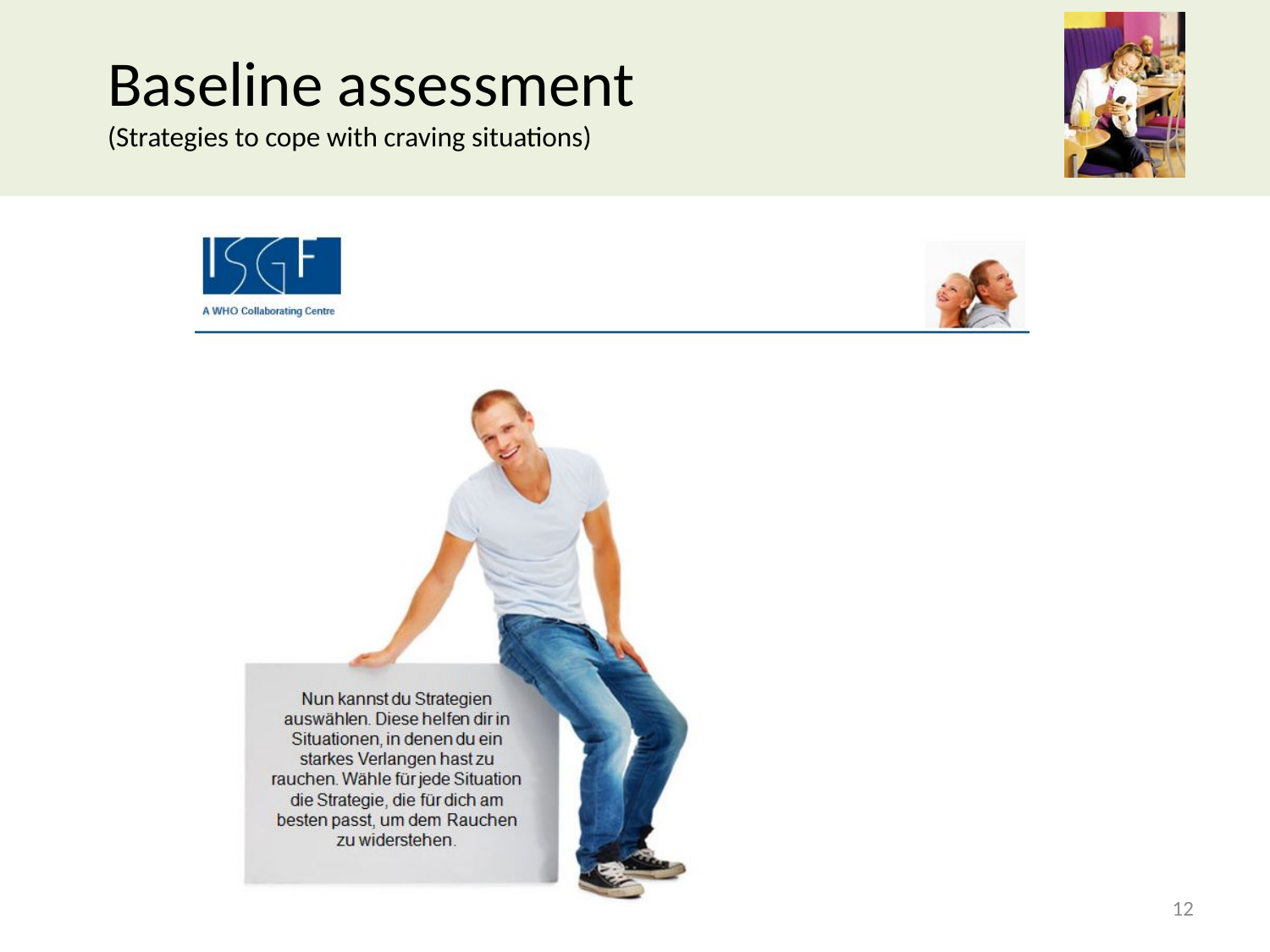

Baseline assessment
(Strategies to cope with craving situations)
12

## Slide 13
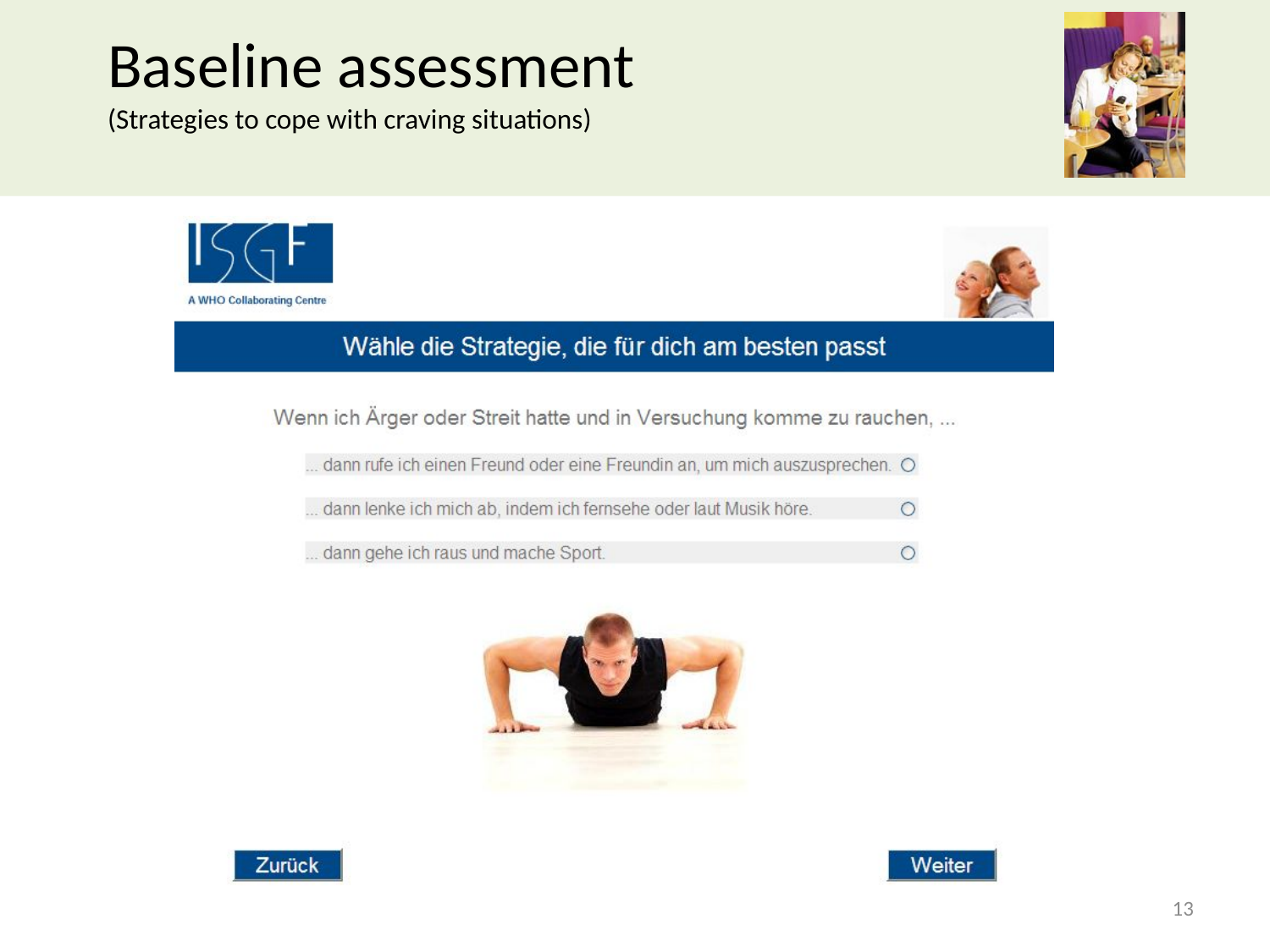

Baseline assessment
(Strategies to cope with craving situations)
13

## Slide 14
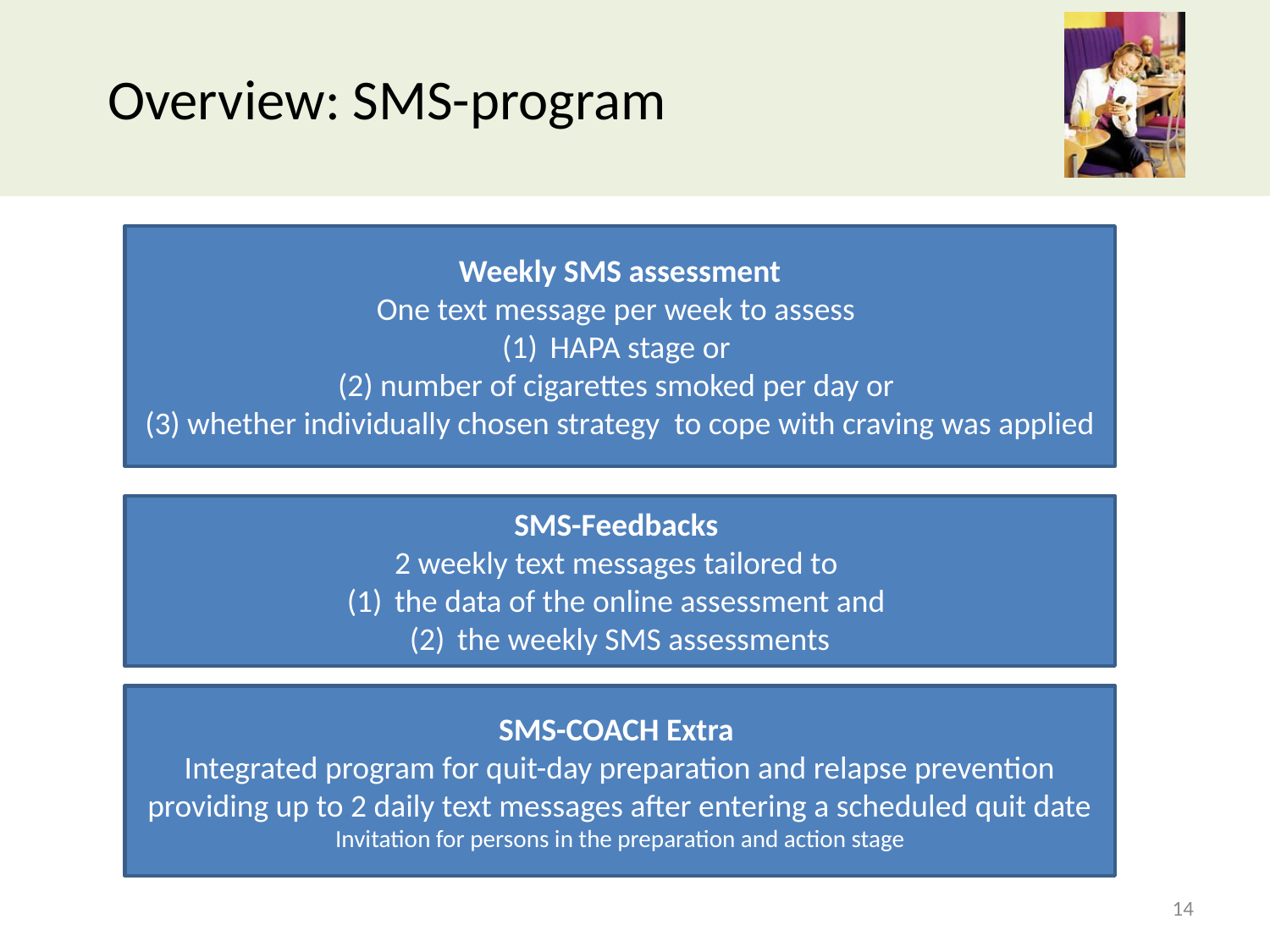

Overview: SMS-program
Weekly SMS assessment
One text message per week to assess
HAPA stage or
(2) number of cigarettes smoked per day or
(3) whether individually chosen strategy to cope with craving was applied
SMS-Feedbacks
2 weekly text messages tailored to
the data of the online assessment and
the weekly SMS assessments
SMS-COACH Extra
Integrated program for quit-day preparation and relapse prevention providing up to 2 daily text messages after entering a scheduled quit date
Invitation for persons in the preparation and action stage
14

## Slide 15
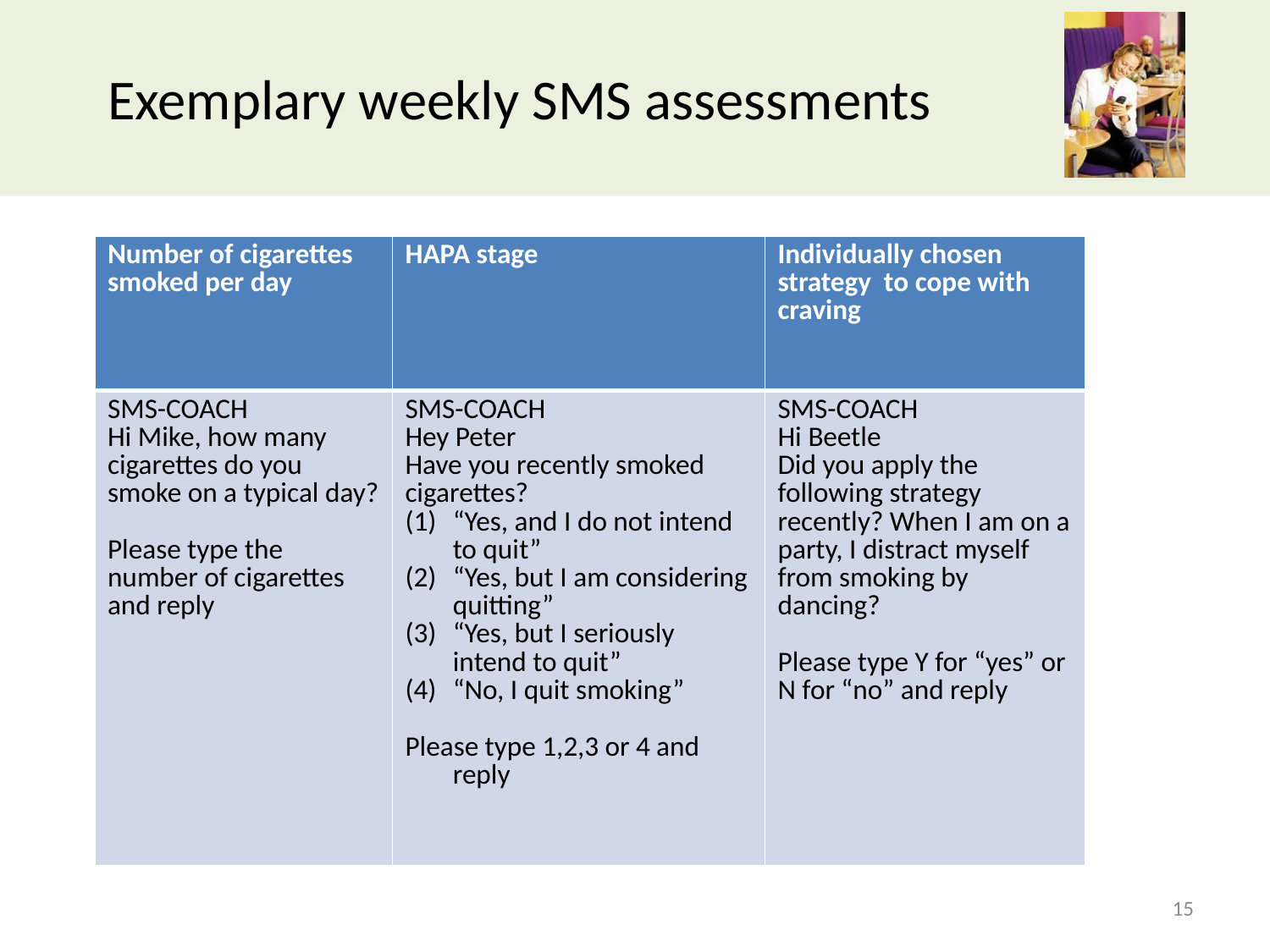

Exemplary weekly SMS assessments
| Number of cigarettes smoked per day | HAPA stage | Individually chosen strategy to cope with craving |
| --- | --- | --- |
| SMS-COACH Hi Mike, how many cigarettes do you smoke on a typical day? Please type the number of cigarettes and reply | SMS-COACH Hey Peter Have you recently smoked cigarettes? “Yes, and I do not intend to quit” “Yes, but I am considering quitting” “Yes, but I seriously intend to quit” “No, I quit smoking” Please type 1,2,3 or 4 and reply | SMS-COACH Hi Beetle Did you apply the following strategy recently? When I am on a party, I distract myself from smoking by dancing? Please type Y for “yes” or N for “no” and reply |
15

## Slide 16
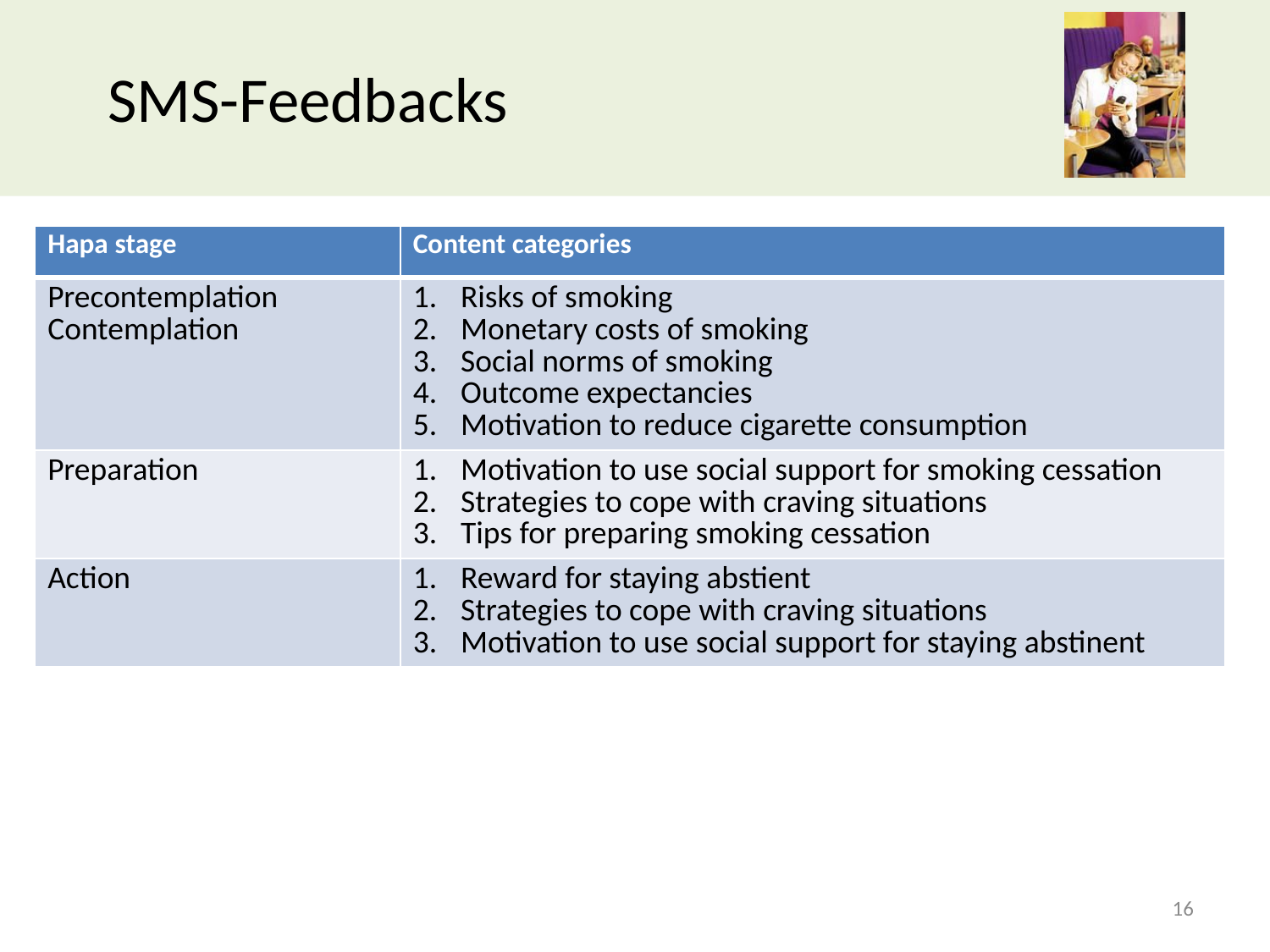

SMS-Feedbacks
| Hapa stage | Content categories |
| --- | --- |
| Precontemplation Contemplation | Risks of smoking Monetary costs of smoking Social norms of smoking Outcome expectancies Motivation to reduce cigarette consumption |
| Preparation | Motivation to use social support for smoking cessation Strategies to cope with craving situations Tips for preparing smoking cessation |
| Action | Reward for staying abstient Strategies to cope with craving situations Motivation to use social support for staying abstinent |
16

## Slide 17
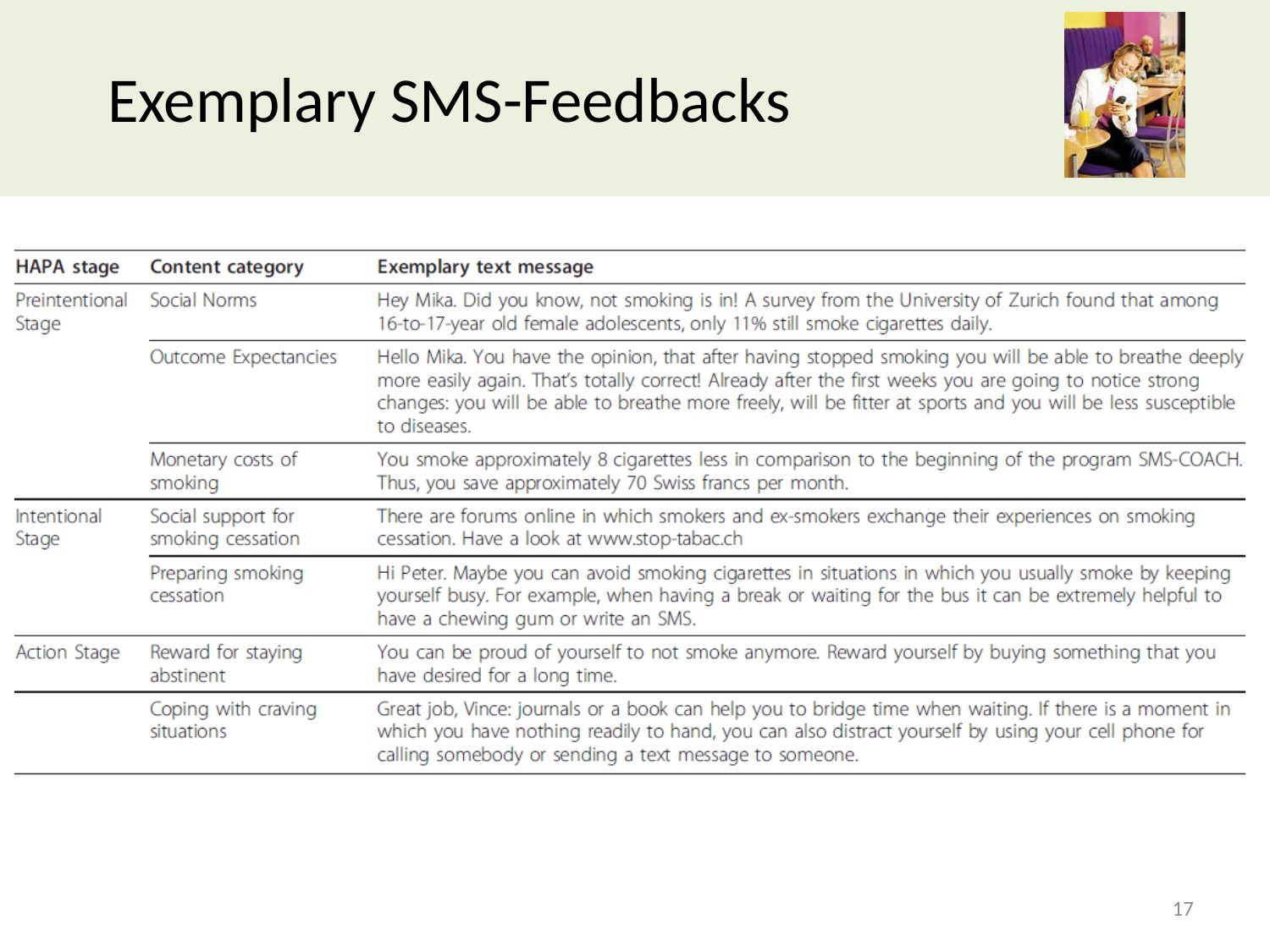

Exemplary SMS-Feedbacks
17
