# Supplementary material for: Efficacy of a Text Message-Based Smoking Cessation Intervention for Young People: A Cluster Randomized Controlled Trial
Source: J Med Internet Res. 2013 Aug 16;15(8):e171. doi: 10.2196/jmir.2636 (PMC3757913; doi:10.2196/jmir.2636)
Supplement: Supplementary file 2 [file jmir_v15i8e171_app2.pdf]

CONSORT-EHEALTH Checklist V1.6.2 Report

(based on CONSORT-EHEALTH V1.6), available at [http://tinyurl.com/consort-ehealth-v1-6].

2636

Date completed

3/20/2013 8:27:00

by

Severin Haug

Efficacy of a Text Messaging (SMS) Based Smoking Cessation Intervention for Young People: A Cluster Randomised Controlled Trial

TITLE

1a-i) Identify the mode of delivery in the title

Title: "Efficacy of a Text Messaging (SMS) Based..."

1a-ii) Non-web-based components or important co-interventions in title

not applicable

1a-iii) Primary condition or target group in the title

Title: "Efficacy of a Text Messaging (SMS) Based Smoking Cessation Intervention for Young People: ..."

ABSTRACT

1b-i) Key features/functionalities/components of the intervention and comparator in the METHODS section of the ABSTRACT

Abstract: "Theoretical background of the intervention was the Health Action Process Approach. Text messages, tailored to demographic and smoking related variables, were sent to the participants of the intervention group over a period of 3 months."

1b-ii) Level of human involvement in the METHODS section of the ABSTRACT

Abstract: "Objectives: To test the efficacy of an individually tailored, fully-automated text messaging (SMS) based intervention for smoking cessation in young people."

1b-iii) Open vs. closed, web-based (self-assessment) vs. face-to-face assessments in the METHODS section of the ABSTRACT

Abstract: "Smoking apprentices were proactively recruited via online screening in vocational school classes."

1b-iv) RESULTS section in abstract must contain use data

Abstract: "A total of 2638 apprentices of 178 vocational school classes in Switzerland participated in the online screening. 1012 persons met the inclusion criteria for study participation and 755 per-sons (74.6%) participated in the study (372 in the intervention group, 383 in the control group). 9 (2.4%) out of 372 participants of the intervention group unsubscribed from the program during the intervention period. 6-months follow-up was gathered in 559 study par-ticipants (74%)."

1b-v) CONCLUSIONS/DISCUSSION in abstract for negative trials

Abstract, Conclusions: "The intervention did not have short-term effects on smoking cessation, however resulted in lower cigarette consumption and further beneficial effects on variables related to smoking cessation within the subgroup of occasional smokers."

INTRODUCTION

2a-i) Problem and the type of system/solution

Introduction: "Tobacco use is a major cause of disease burden and the single most preventable cause of death in the world [1]. A survey among 15 and 16 year old adolescents covering 36 European countries revealed that the smoking prevalence rates of 28% having used cigarettes during the past 30 days remained rather stable over the last 4 years [2]. Smoking continues to be a serious problem, particularly in adolescents and young adults with lower educational level [3].
There is limited evidence of smoking cessation interventions demonstrating efficacy in young people [4, 5]."

"Mobile phone text messaging (SMS) is very popular among adolescents and young adults and has the potential to deliver smoking cessation support to large proportions of populations. Among 12-to-19-year-old adolescents from Switzerland 98% owned a mobile phone in the year 2010; use of the mobile phone was the most frequent leisure time activity in this popula-tion group [12]. Reading and sending SMS were the most frequent activities when using the mobile phone [12].
By use of expert system technology that provides information based on individual demo-graphic or smoking-related characteristics, electronic communication technology can be a viable, time- and cost-saving alternative to interpersonal counselling [13]. Particularly SMS provides opportunities for individualised and interactive information delivery that may easily be accessed, independent of time and place."

2a-ii) Scientific background, rationale: What is known about the (type of) system

Introduction: "To date, neither randomised controlled trials testing the efficacy of smoking cessation inter-ventions employing SMS in adolescents and young adults nor trials testing the efficacy of SMS interventions in proactively recruited smokers have been reported. Within three pilot studies in which young adult smokers, irrespective of their motivation to quit, were proac-tively invited to an SMS based smoking cessation intervention, high participation and reten-tion proportions could be achieved [16-18]."

METHODS

3a) CONSORT: Description of trial design (such as parallel, factorial) including allocation ratio

Introduction: "Within the present cluster randomised trial, we tested the efficacy of an SMS based interven-tion for smoking cessation in a sample of proactively recruited apprentices with different motivation to quit."

3b) CONSORT: Important changes to methods after trial commencement (such as eligibility criteria), with reasons

Methods: Study design:
"The study was implemented as described in the study protocol [19] with the following modi-fications: (1) due to smaller class sizes as expected and time restrictions, we could not reach the targeted sample size of 910 study participants but merely 755 study participants. (2) Self-efficacy for smoking cessation could not be assessed at follow-up and used as secondary out-come measures because the rating scale to assess this variable [20] could not be applied within the telephone interviews conducted at follow-up. (3) Nicotine dependence could not be calculated for occasional smokers using the Heaviness of Smoking Index [21]. Therefore, we used number of cigarettes smoked per day as an indicator of nicotine dependence and as outcome variable."

3b-i) Bug fixes, Downtimes, Content Changes

not applicable

4a) CONSORT: Eligibility criteria for participants

Methods: Participant recruitment and baseline assessment:
"Inclusion criteria for study participation were (1) daily or occasional cigarette smoking (at least 4 cigarettes in the preceding month and at least one cigarette during the preceding week) and (2) ownership of a mobile phone."

4a-i) Computer / Internet literacy

4a-ii) Open vs. closed, web-based vs. face-to-face assessments:

Methods: Participant recruitment and baseline assessment:
"Study participants were recruited by study assistants (graduate students of psychology). The study assistants invited all apprentices from a school class to participate in an online health survey during a regular school lesson, reserved for health education..."

"Afterwards the apprentices were invited to fill in an online screening. The screening included..."

4a-iii) Information giving during recruitment

Methods: Participant recruitment and baseline assessment:

"The study assistants invited all apprentices from a school class to participate in an online health survey during a regular school lesson, reserved for health education. Furthermore, they informed the apprentices that some persons will be invited to participate in a study testing the efficacy of a text messaging intervention for health promotion. To decrease reporting bias the study assistants did not provide more information about the purpose of the study before the screening of eligibility criteria was completed."

4b) CONSORT: Settings and locations where the data were collected

Methods: Participant recruitment and baseline assessment:
"Smoking apprentices were recruited at vocational schools in Switzerland. Contact teachers for addiction prevention or headmasters of 57 vocational schools in German speaking regions of Switzerland were invited to participate with some of their classes in a study testing the effi-cacy of a text messaging based smoking cessation program."

4b-i) Report if outcomes were (self-)assessed through online questionnaires

Methods: Follow-up measures:
"Computer assisted telephone interviews were conducted at the six months follow-up assess-ment by trained interviewers. The following outcome variables were assessed during this interview:..."

4b-ii) Report how institutional affiliations are displayed

5) CONSORT: Describe the interventions for each group with sufficient details to allow replication, including how and when they were actually administered

5-i) Mention names, credential, affiliations of the developers, sponsors, and owners

5-ii) Describe the history/development process

Methods: Intervention:

"The text messaging intervention SMS-COACH was fully automated and based on Internet technology using a LAMP system (Linux, Apache, MySQL, and PHP). The program used in the present study was an extended and modified version of a previous version which had suc-cessfully been tested in pilot studies [16-18]. All incoming and outgoing text messages were automatically recorded. Incoming messages were analyzed immediately 24 hr/day."

5-iii) Revisions and updating

5-iv) Quality assurance methods

5-v) Ensure replicability by publishing the source code, and/or providing screenshots/screen-capture video, and/or providing flowcharts of the algorithms used

5-vi) Digital preservation

5-vii) Access

Methods: Participant recruitment and baseline assessment:

"Smoking apprentices were recruited at vocational schools in Switzerland..."
"The study assistants invited all apprentices from a school class to participate in an online health survey during a regular school lesson, reserved for health education..."
"After receiving informed consent online, all study participants were invited to choose a username and to provide their mobile phone number."

...reimbursement of the equivalent of 0.80 Euro was offered to the participants of the intervention group for each SMS response to the weekly SMS assessments within the program."

5-viii) Mode of delivery, features/functionalities/components of the intervention and comparator, and the theoretical framework

Methods: Intervention:

"Intervention
Technological background
The text messaging intervention SMS-COACH was fully automated and based on Internet technology using a LAMP system (Linux, Apache, MySQL, and PHP). The program used in the present study was an extended and modified version of a previous version which had suc-cessfully been tested in pilot studies [16-18]. All incoming and outgoing text messages were automatically recorded. Incoming messages were analyzed immediately 24 hr/day.

Theoretical background
Primarily, the program was based on the Health Action Process Approach (HAPA) [20]. This health behaviour model suggests a distinction between motivation processes resulting in goal setting and volition processes leading to the actual health behaviour. The approach combines three 'nonactive' stages (Precontemplation, Contemplation, Preparation) and two 'active' stages (Action, Maintenance). Within the initial two stages, outcome expectancies, risk per-ception, and perceived self-efficacy are seen as important social-cognitive predictors in order to develop an intention to act. Within the subsequent intentional stage (Preparation), planning processes are crucial in order to achieve the desired action. Once an action has been initiated, self-regulatory skills are important to maintain the health behaviour. In addition to the HAPA we used intervention elements derived from the Social Norms Approach [23] and implemen-tation intentions , which are if-then plans that link situational cues with responses that are effective in attaining a desired outcome [24].

Intervention elements
The intervention program consisted of (1) an online assessment of the individual smoking behaviour and smoking related attitudes, (2) a weekly SMS-assessment of smoking-related target behaviours, (3) two weekly text messages tailored to the data of the online and the SMS-assessments, and (4) an integrated quit day preparation and relapse prevention program.

Online baseline assessment
Beyond the screening questions and the above mentioned smoking related variables, which were assessed in both study groups within the baseline assessment, participants of the inter-vention group additionally received online questions assessing (1) outcome expectancies of smoking cessation, (2) situations or circumstances in which craving for cigarettes usually oc-curs, (3) alternative strategies to handle these craving situations, and (4) costs per cigarette package.

Weekly SMS assessment
During the 3-month intervention period, participants of the intervention group received one text message per week for the assessment of smoking related target behaviour. This question could easily be answered by typing a single letter or number, and using the reply function of the mobile phone. The weekly SMS assessment question was sent at a fixed point in time each week (6 p.m. at the weekday of registration for the program). The content of the question de-pended on the HAPA stage as well as on the number of the intervention week.
For all participants, the HAPA stage was assessed in even weeks by the question: "Have you recently smoked cigarettes?" with the following response options (1) "Yes, and I do not in-tend to quit" (Precontemplation), (2) "Yes, but I am considering to quit" (Contemplation), (3) "Yes, but I seriously intend to quit" (Preparation), or (4) "No, I quit smoking" (Action). This question assessed both smoking status and intention to quit over time and the responses to this question allowed tailoring the SMS feedbacks according to the current HAPA stage [25].
In odd weeks, we assessed the number of cigarettes smoked per day or week (depending on smoking status: daily/occasionally) in smokers of the preintentional stages (Precontemplation and Contemplation); and, we assessed whether smokers in the intention or action stage ap-plied the individually chosen strategies to cope with craving situations (e.g. "Did you apply the following strategy recently? When I am on a party, I distract myself from smoking by dancing.").

Individually tailored text messages
On the first level, the text messages were tailored to the HAPA stage. Persons in the preinten-tional stages received text messages addressing (1) risks of smoking, (2) monetary costs of smoking, (3) social norms of smoking, (4) outcome expectancies, and (5) motivation to reduce the number of cigarettes smoked per day (daily smokers) or week (occasional smokers). Persons in the intentional stage received text messages which (1) motivated to use social sup-port for smoking cessation, (2) provided strategies to cope with craving situations, and (3) provided tips for preparing smoking cessation (e.g. reducing the number of cigarettes, identify craving situations). Persons in the action stage received text messages (1) motivating to reward themselves for staying abstinent, (2) providing strategies to cope with craving situations, and (3) motivating to use social support for staying abstinent.
On the second level, the text messages were tailored according to the individual information provided at the baseline assessment as well as through the weekly SMS assessments. Exem-plary text messages are displayed in the study protocol for this trial [19].

Integrated program for quit day preparation and relapse prevention
Persons in the preparation and action stage had the possibility to additionally participate in an integrated program for quit day preparation and relapse prevention. Program participants in these stages were informed biweekly about this option. After entering a scheduled quit date, this program provided up to two daily text messages (weeks -1 to +1: two daily SMS; weeks +2 and +3: one daily text message) in order to prepare for the quit day and to prevent relapse afterwards.

Control group
Study participants of the assessment only control group did not receive any of the above de-scribed intervention elements of the SMS-COACH.
5-ix) Describe use parameters
Methods: Intervention:

"The intervention program consisted of (1) an online assessment of the individual smoking behaviour and smoking related attitudes, (2) a weekly SMS-assessment of smoking-related target behaviours, (3) two weekly text messages tailored to the data of the online and the SMS-assessments, and (4) an integrated quit day preparation and relapse prevention program."

"Integrated program for quit day preparation and relapse prevention
Persons in the preparation and action stage had the possibility to additionally participate in an integrated program for quit day preparation and relapse prevention. Program participants in these stages were informed biweekly about this option. After entering a scheduled quit date, this program provided up to two daily text messages (weeks -1 to +1: two daily SMS; weeks +2 and +3: one daily text message) in order to prepare for the quit day and to prevent relapse afterwards."
5-x) Clarify the level of human involvement

5-xi) Report any prompts/reminders used

Beyond the weekly SMS assessment and the weekly provided feedback messages, we did not apply additional prompts or reminders.

5-xii) Describe any co-interventions (incl. training/support)

we did not provide any co-interventions

6a) CONSORT: Completely defined pre-specified primary and secondary outcome measures, including how and when they were assessed

Methods: Follow-up measures:

"Computer assisted telephone interviews were conducted at the six months follow-up assess-ment by trained interviewers. The following outcome variables were assessed during this in-terview: (1) smoking status, (2) 7-day and (3) 30-day smoking abstinence, (4) mean number of cigarettes smoked per day, (5) stage of change according to the HAPA and (6) quit attempts within the past six months preceding the follow-up. The main outcome criterion was 7-day point prevalence smoking abstinence."

6a-i) Online questionnaires: describe if they were validated for online use and apply CHERRIES items to describe how the questionnaires were designed/deployed

6a-ii) Describe whether and how "use" (including intensity of use/dosage) was defined/measured/monitored

Methods: Program participation and program use:

"To evaluate the acceptance of the program, we analyzed the log files of the SMS system in which the number and content of incoming and outgoing text messages were recorded. The number of responses to the weekly SMS assessments and the number of program participants who unsubscribed from the program (program attrition) were examined. At follow-up, we also assessed an aspect of the usage of the SMS messages by asking the participants whether they (1) read through the SMS feedback messages thoroughly, (2) took only a short look at the feedback messages, or (3) did not read the feedback messages."

6a-iii) Describe whether, how, and when qualitative feedback from participants was obtained

Methods: Intervention:

"The program used in the present study was an extended and modified version of a previous version which had successfully been tested in pilot studies [16-18]."

6b) CONSORT: Any changes to trial outcomes after the trial commenced, with reasons

Methods: Study design:

"The study was implemented as described in the study protocol [19] with the following modifications: (1) due to smaller class sizes as expected and time restrictions, we could not reach the targeted sample size of 910 study participants but merely 755 study participants. (2) Self-efficacy for smoking cessation could not be assessed at follow-up and used as secondary out-come measures because the rating scale to assess this variable [20] could not be applied within the telephone interviews conducted at follow-up. (3) Nicotine dependence could not be calculated for occasional smokers using the Heaviness of Smoking Index [21]. Therefore, we used number of cigarettes smoked per day as an indicator of nicotine dependence and as out-come variable."

7a) CONSORT: How sample size was determined

7a-i) Describe whether and how expected attrition was taken into account when calculating the sample size

Methods: Sample size calculation:

"Based on results of a study which tested the efficacy of telephone counselling for smoking cessation in high school students [8], we expected an 8% difference in 7-day point prevalence abstinence rates between the intervention and the control condition at 6-months follow up assessment (25% versus 17% respectively). To achieve power at 0.80 with a significance level of 0.05, using a Chi-Square Test, a sample size of n=406 in each study group was necessary to show this difference. As apprentices are nested within school classes, we additionally needed to consider a potential design effect of 1.12 (average cluster size n=7; intra-cluster correlation coefficient: 0.02) which resulted in a required sample size of n=455 per study group."

7b) CONSORT: When applicable, explanation of any interim analyses and stopping guidelines

not applicable

8a) CONSORT: Method used to generate the random allocation sequence

Methods: Randomisation and allocation concealment:

"To avoid spill-over effects within school classes, we used cluster-randomisation with school class as randomisation unit. Due to the heterogeneity of apprentices in the different vocational schools (e.g. concerning gender or professions), we used separate randomisation lists for each vocational school (stratified randomisation). Furthermore, to approximate equality of sample sizes in the study groups, we used block randomisation with computer generated randomly permuted blocks of 4 cases [22]."

8b) CONSORT: Type of randomisation; details of any restriction (such as blocking and block size)

Methods: Randomisation and allocation concealment:

"To avoid spill-over effects within school classes, we used cluster-randomisation with school class as randomisation unit. Due to the heterogeneity of apprentices in the different vocational schools (e.g. concerning gender or professions), we used separate randomisation lists for each vocational school (stratified randomisation). Furthermore, to approximate equality of sample sizes in the study groups, we used block randomisation with computer generated randomly permuted blocks of 4 cases [22]."

9) CONSORT: Mechanism used to implement the random allocation sequence (such as sequentially numbered containers), describing any steps taken to conceal the sequence until interventions were assigned

Methods: Randomisation and allocation concealment:

"To avoid spill-over effects within school classes, we used cluster-randomisation with school class as randomisation unit. Due to the heterogeneity of apprentices in the different vocational schools (e.g. concerning gender or professions), we used separate randomisation lists for each vocational school (stratified randomisation). Furthermore, to approximate equality of sample sizes in the study groups, we used block randomisation with computer generated randomly permuted blocks of 4 cases [22]."

10) CONSORT: Who generated the random allocation sequence, who enrolled participants, and who assigned participants to interventions

The random allocation sequence was computer generated by the principal investigator.

The study assistants who conducted the baseline assessment in the vocational schools were blinded concerning group allocation of the school classes. Also, group allocation was not re-leased to study participants until having provided informed consent, username, mobile phone number, and baseline data of the smoking-related variables. Participants were enrolled online and assigned to the study group based on computer generated randomly permuted blocks of 4 cases"

11a) CONSORT: Blinding - If done, who was blinded after assignment to interventions (for example, participants, care providers, those assessing outcomes) and how

11a-i) Specify who was blinded, and who wasn't

Methods: Participant recruitment and baseline assessment:

"Afterwards, study participants of the intervention group received further information about the operation of the program. Control group participants were informed that they were assigned to the control group and could not participate in the SMS-program."

"Randomisation and allocation concealment

...The study assistants who conducted the baseline assessment in the vocational schools were blinded concerning group allocation of the school classes. Also, group allocation was not released to study participants until having provided informed consent, username, mobile phone number, and baseline data of the smoking-related variables. The study assistants who conducted the computer assisted telephone interviews at follow-up were blinded concerning group allocation when assessing the primary and secondary outcome measures."

11a-ii) Discuss e.g., whether participants knew which intervention was the "intervention of interest" and which one was the "comparator"

11b) CONSORT: If relevant, description of the similarity of interventions

not applicable

12a) CONSORT: Statistical methods used to compare groups for primary and secondary outcomes

Methods: Data analyses:

"We used regression models to test the efficacy of the intervention on the different outcome measures. Logistic regression models were applied for the binary outcome variables (7-day and 30-day point prevalence smoking abstinence), negative binomial regression models were applied for count data (number of cigarettes smoked per day), ordinal logistic regression models were used for ordinal data (stage of change) and multinomial logistic regression mod-els were used for categorical outcomes (smoking status). To control for baseline differences, we additionally added the respective baseline variables as covariates to the regression models."

"Given the clustered nature of the data (apprentices within school classes) we com-puted robust variance estimators for all regression models, using the svy command of STATA."

12a-i) Imputation techniques to deal with attrition / missing values

Methods: Data analyses:

"We conducted both (1) complete case analyses (CCA) considering all study participants with available follow-up data and (2) intention to treat (ITT) analyses. For the ITT analyses we applied the multiple imputations procedure (MICE) of STATA, which imputed missing fol-low-up data using all available baseline variables (demographic, health- and smoking related variables)."

12b) CONSORT: Methods for additional analyses, such as subgroup analyses and adjusted analyses

Methods: Data analyses:

"To control for baseline differences, we additionally added the respective baseline variables as covariates to the regression models."

"Due to significant baseline differences between the study groups, particularly in the percent-age of occasional and daily smokers, we additionally conducted outcome analyses, separately for occasional and daily smokers."

RESULTS

13a) CONSORT: For each group, the numbers of participants who were randomly assigned, received intended treatment, and were analysed for the primary outcome

Results

Study participation

Figure 1

"Due to smaller class sizes as expected and time restrictions, we could not reach the targeted sample size of 910 study participants. Figure 2 presents the flow of the study participants. At the time of the online screening assessment in 178 school classes, a total of 2657 apprentices were present. Among them, 2638 (99.3%) agreed to participate. Of these, 1012 persons met the inclusion criteria for study participation and 755 persons (74.6%) participated in the study. Of all school classes, 90 including 372 apprentices were randomly assigned to the intervention group, 88 school classes including 383 apprentices were assigned to the control group. Follow-up assessments were realized in 287 (77.2%) of 372 study participants of the intervention group and in 272 (71.0%) of 383 study participants of the control group."

13b) CONSORT: For each group, losses and exclusions after randomisation, together with reasons

see flow diagram figure 1

13b-i) Attrition diagram

Results

Program attrition and program use

"During the program, which lasted for 3 months, 9 (2.4%) out of the 372 participants of the intervention group unsubscribed from the program.
The mean number of replies to the weekly SMS assessments was 6.5 (SD=3.7). No reply was sent by 34 persons (9.1%) and all 11 replies were sent by 55 persons (14.8%)."

14a) CONSORT: Dates defining the periods of recruitment and follow-up

Methods: Study design:

"The trial was undertaken in Switzerland and participants were recruited between October 2011 and May 2012; 6-month follow-ups were conducted between April 2012 and December 2012; the protocol was published on 19/01/2012 [19]."

14a-i) Indicate if critical "secular events" fell into the study period

not applicable

14b) CONSORT: Why the trial ended or was stopped (early)

Methods

Study design

"... (1) due to smaller class sizes as expected and time restrictions, we could not reach the targeted sample size of 910 study participants but merely 755 study participants."

15) CONSORT: A table showing baseline demographic and clinical characteristics for each group

Results: Sample characteristics:

Table 1.
15-i) Report demographics associated with digital divide issues
Results: Sample characteristics:

Table 1.
16a) CONSORT: For each group, number of participants (denominator) included in each analysis and whether the analysis was by original assigned groups
16-i) Report multiple “denominators” and provide definitions
Results: Program efficacy:

see Tables 2 and 3.

"Quit attempts
Based on complete case data of the total sample, 98 (36.3%) of 270 persons from the control group and 125 (43.7%) of 286 persons from the intervention group indicated that they made a quit attempt within six months preceding the follow-up (CCA: OR 1.17, 95% CI 0.81-1.71, P=.40; ITT: OR .1.23 95% CI 0.85-1.78, P=.27). In baseline occasional smokers, 12 (43.1%) of 51 persons from the control group and 62 (68.9%) of 90 persons of the intervention group indicated a quit attempt (CCA: OR 2.79, 95% CI 1.36- 5.73, P=.006; ITT: OR 2.47, 95% CI 1.03-5.94, P=.04). Using the subgroup of baseline daily smokers, 76 (34.7%) of 219 persons from the control group and 63 (32.1%) of 196 persons of the intervention group indicated a quit attempt (CCA: OR 0.87, 95% CI 0.55-1.37, P=.54; ITT: 1.00, 95% CI 0.67-1.48, P=.98)."
16-ii) Primary analysis should be intent-to-treat
See section: Program efficacy:

Both ITT and CCA were performed.
17a) CONSORT: For each primary and secondary outcome, results for each group, and the estimated effect size and its precision (such as 95% confidence interval)
See section: Results: Program efficacy

Table 2.
17a-i) Presentation of process outcomes such as metrics of use and intensity of use
Results:

Program attrition and program use

"During the program, which lasted for 3 months, 9 (2.4%) out of the 372 participants of the intervention group unsubscribed from the program.
The mean number of replies to the weekly SMS assessments was 6.5 (SD=3.7). No reply was sent by 34 persons (9.1%) and all 11 replies were sent by 55 persons (14.8%).
Out of the 287 persons with valid follow-up data, 271 (94.4%) indicated that they regularly read the SMS messages. Of these, 204 (75.3%) indicated that they “read the SMS messages thoroughly” while 67 persons (24.7%) reported that they “took a short look at the feedback messages”."
17b) CONSORT: For binary outcomes, presentation of both absolute and relative effect sizes is recommended
See results section: Program efficacy: Smoking abstinence

Table 2

"Quit attempts
Based on complete case data of the total sample, 98 (36.3%) of 270 persons from the control group and 125 (43.7%) of 286 persons from the intervention group indicated that they made a quit attempt within six months preceding the follow-up (CCA: OR 1.17, 95% CI 0.81-1.71, P=.40; ITT: OR .1.23 95% CI 0.85-1.78, P=.27). In baseline occasional smokers, 12 (43.1%) of 51 persons from the control group and 62 (68.9%) of 90 persons of the intervention group indicated a quit attempt (CCA: OR 2.79, 95% CI 1.36- 5.73, P=.006; ITT: OR 2.47, 95% CI 1.03-5.94, P=.04). Using the subgroup of baseline daily smokers, 76 (34.7%) of 219 persons from the control group and 63 (32.1%) of 196 persons of the intervention group indicated a quit attempt (CCA: OR 0.87, 95% CI 0.55-1.37, P=.54; ITT: 1.00, 95% CI 0.67-1.48, P=.98)."

18) CONSORT: Results of any other analyses performed, including subgroup analyses and adjusted analyses, distinguishing pre-specified from exploratory
Yes, subgroup analyses for occasional and daily smokers are integrated int the section "results": "program efficacy"
18-i) Subgroup analysis of comparing only users
was not applied
19) CONSORT: All important harms or unintended effects in each group
no harms or unintended effects were observed within the study
19-i) Include privacy breaches, technical problems

19-ii) Include qualitative feedback from participants or observations from staff/researchers

DISCUSSION
20) CONSORT: Trial limitations, addressing sources of potential bias, imprecision, multiplicity of analyses
20-i) Typical limitations in ehealth trials

Discussion: Limitations

"Several limitations must be noted. First, smoking status was assessed by self-report and was not biochemically verified. However, it is expected that a potential over-reporting of smoking abstinence will be independent of the study condition. Furthermore, based on recommenda-tions by the Society for Research on Nicotine and Tobacco, there are circumstances under which the added precision gained by biological validation is offset in such a way that its use is not required and may not be desirable [38]. Examples given are population-based studies with low demand on smokers to quit, e.g. interventions with limited face-to-face contact and stud-ies where the optimal data collection methods are through mail, telephone or Internet. A sec-ond limitation is that we only investigated short-term effects of the program. Longer follow-up assessments might provide different results. However, both of these limitations resulted in a lower expenditure of time for the study participants and a greater proximity to prevention practice. Therefore, they allowed a better estimation of the participation rate in the program that might be expected under routine intervention conditions. A third study limitation is its lack of statistical power, particularly for the subgroup analyses."

21) CONSORT: Generalisability (external validity, applicability) of the trial findings

21-i) Generalizability to other populations

Discussion: Conclusions:

"Both the baseline assessment and the registration for the SMS program are possible from every computer with Internet access and take only approximately ten minutes. Therefore, the program could be easily and economically implemented within school classes, within web-based smoking cessation tools or within prevention campaigns."

21-ii) Discuss if there were elements in the RCT that would be different in a routine application setting

not applicable

22) CONSORT: Interpretation consistent with results, balancing benefits and harms, and considering other relevant evidence

22-i) Restate study questions and summarize the answers suggested by the data, starting with primary outcomes and process outcomes (use)

Discussion

The study aimed at testing the efficacy of an SMS based intervention for smoking cessation in a sample of proactively recruited apprentices with different motivation to quit. The study re-vealed three main findings: (1) a large percentage of smoking apprentices at vocational schools could be reached by the program, (2) program attrition was low and (3) results from the 6-months follow-up showed that program participation resulted in lower cigarette consumption. No short-term effect of the intervention on smoking abstinence rates was found.

22-ii) Highlight unanswered new questions, suggest future research

Other information

23) CONSORT: Registration number and name of trial registry

Methods

Study design

"A two-arm cluster-randomised controlled trial (ISRCTN19739792 assigned on 20/05/2011)..."

24) CONSORT: Where the full trial protocol can be accessed, if available

Methods

Study design

"...the study protocol was published on 19/01/2012 [19]."

25) CONSORT: Sources of funding and other support (such as supply of drugs), role of funders

"Acknowledgements

Funding for this study was provided by the Swiss Tobacco Prevention Fund (No 10.007207). The funding institution had no role in the design or conduct of the study, the collection, man-agement, analysis, or interpretation of the data, or the preparation, review or approval of the manuscript."

X26-i) Comment on ethics committee approval

Methods: Study design:

"The study protocol was approved by the Local Ethics Committee of the Canton of Zurich, Switzerland (Date of approval: 15 March, 2011; No: KEK-StV-Nr. 05/11). The trial was exe-cuted in compliance with the Helsinki Declaration."

x26-ii) Outline informed consent procedures

X26-iii) Safety and security procedures

X27-i) State the relation of the study team towards the system being evaluated

"Competing interests

Single authors (SH, CM) were also involved in the development of the intervention."
